# Switching On/Off of a Solvent Coordination in a Au(I)–Pb(II) Complex: High Pressure and Temperature as External Stimuli

**DOI:** 10.1021/acs.inorgchem.3c01130

**Published:** 2023-06-16

**Authors:** Sonia Moreno, Nicola Casati, María Rodríguez-Castillo, Miguel Monge, M. Elena Olmos, José M. López-de-Luzuriaga

**Affiliations:** † Departamento de Química, Centro de Investigación en Síntesis Química (CISQ), Complejo Científico-Tecnológico, Universidad de La Rioja, 26006 Logroño, Spain; ‡ Laboratory for Synchrotron Radiation−Condensed Matter, Paul Scherrer Institute (PSI), WLGA/229 Forschungsstrasse 111, 5232 Villigen, Switzerland

## Abstract

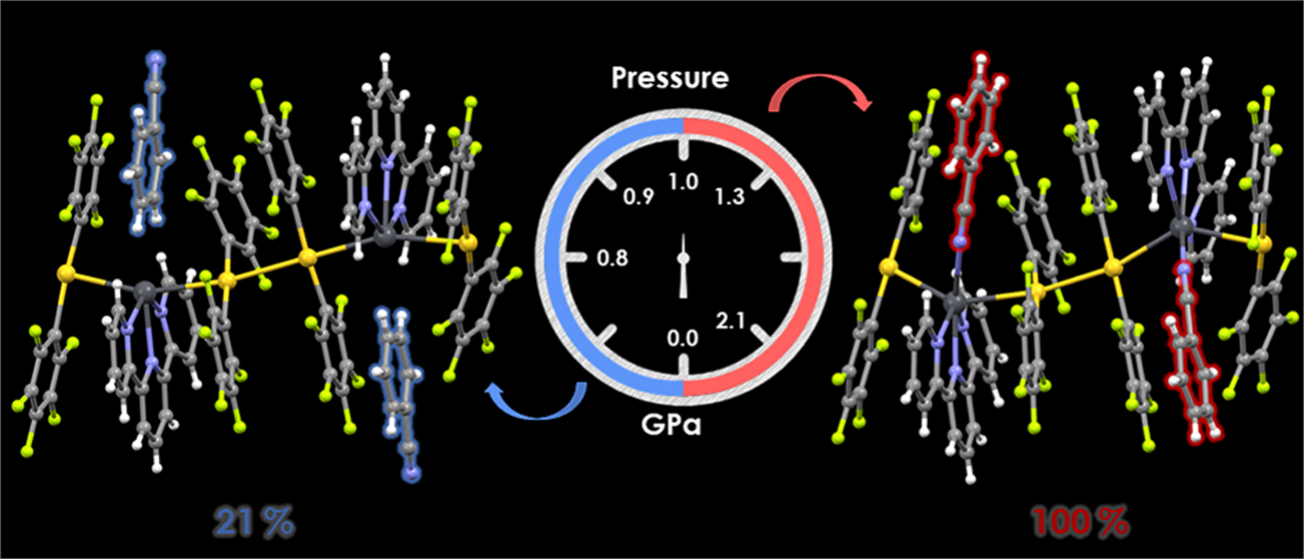

The benzonitrile solvate {[{Au(C_6_F_5_)_2_}_2_{Pb(terpy)}]·NCPh}*_n_* (**1**) (terpy = 2,2′:6′,2″-terpyridine) displays reversible reorientation and coordination of the benzonitrile molecule to lead upon external stimuli. High-pressure X-ray diffraction studies between 0 and 2.1 GPa reveal a 100% of conversion without loss of symmetry, which is totally reversible upon decompression. By variable-temperature X-ray diffraction studies between 100 and 285 K, a partial coordination is achieved.

## Abstract 2

The application of external stimuli such as increasing pressure or lowering temperature causes an increase in the coordination number of lead centers in a heterometallic Au(I)···Pb(II) complex, this process being completely reversible. The coordination/de-coordination change largely affects the NIR-emissive properties.

## Introduction

Researchers have shown in the last two decades a great interest by thermo- or pressure-sensitive materials, constituting a highly active field of research.^1−3^ This interest is prompted by the promising perspectives that these studies could generate for applications in innovative smart materials or engineering devices.^4^

On the one hand, high-pressure studies of a diverse variety of compounds have allowed us to explore changes in specific physical phenomena, such as polymorphism, magnetic behavior, conductivity, and piezochromism.^5−7^ Linked to these physical phenomena is the underlying modification of the structural features of the materials. It has been shown that high pressure can alter intermolecular interactions, such as hydrogen bonds, modify molecular conformations, and alter bond distances and angles, coordination number, or ordering,^8,9^ being in some but not all cases reversible when the material is decompressed. In the case of metal-organic frameworks (MOFs) and zeolites, moreover, hyperfilling and reaction incorporating the pressure media have also been reported.^10,11^

In the particular case of new bonding formation, examples reported in the literature are far from abundant, and they are usually accompanied by a phase transition in the crystal.^12−14^ Coordination of ligands to metal centers upon increased pressures is among the most reported high-pressure X-ray diffraction studies.^15−22^

On the other hand, low-temperature X-ray diffraction measurements have traditionally been employed to measure accurate diffraction intensities and refine models to remove thermal diffuse scattering and anharmonicity that affect severely at occasions the overall data quality. Nevertheless, sometimes, experiments at different temperatures provoke significant changes in the structure. Thus, for instance, the slight shift of a semibridging carbonyl group in the complex [PPN][FeCo(CO)_8_] to a more symmetrical position between the metals when the temperature is lowered down to 28 K;^23^ or the dehydration that a zinc-containing MOF undergoes *via* single-crystal-to-single-crystal transformation;^24^ or even polymorphism in a series of examples have been described, accompanied in all cases by a phase transition.^25^

In the case of complexes displaying unsupported intermetallic interactions, the most remarkable effect of lowering the temperature or increasing the pressure is a mutual approach of the metal centers as a consequence of the compression of the crystal, although there are also some examples in which, due to the steric repulsion of rearranged ligands, the metallic distances experience an elongation.^26^ When closed-shell metal interactions appear, this effect leads to an appreciable change in the luminescence properties that this type of complex usually exhibits.^27^ Nevertheless, in most cases, ligands behave almost as spectators, with slight modifications of bond lengths or dispositions and, most of the times, without contributing additionally related to the property that shows the complex under the external stimuli.^28^

In this paper, we describe a complex that reversibly coordinates a molecule (benzonitrile) of its solvate to a metal center under pressure without a change of symmetry and the study of the relationship between coordination of this solvent and temperature by X-ray diffraction measurements. Optical properties can be tuned thanks to this ligand reorientation, and computational studies confirm this process.

## Results and Discussion

### Synthesis and Characterization

Complex [{Au(C_6_F_5_)_2_}_2_{Pb(terpy)}]*_n_* is obtained according to a published procedure.^29^ Studies in this laboratory show that this complex is able to incorporate several solvent molecules thanks to the presence of cavities in its structure (Figure S1). We have studied in this work pressure and temperature changes with the benzonitrile derivative, and we have also observed other small solvate molecule incorporations that provoke changes in their optical properties and that are currently under study.

**Scheme 1:**
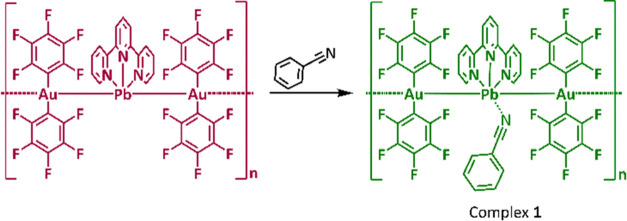
Synthesis of Complex **1**

Thus, a solution of [{Au(C_6_F_5_)_2_}_2_{Pb(terpy)}]*_n_* in benzonitrile was stirred for 10 min, and the incorporation of this molecule into the structure of the starting compound was observed since a change in the color of the solid was detected (Figure S2) from a red solid to a green one with stoichiometric {[{Au(C_6_F_5_)_2_}_2_{Pb(terpy)}]·NCPh}*_n_* (**1**), whose analytical and spectroscopic data agree with the proposed stoichiometry (Scheme 1).

Its IR spectrum (Figure S3) shows, among others, the absorption bands related to the presence of [Au(C_6_F_5_)_2_]^−^ units at 1504, 955, and 770 cm^–1^, as well as those due to the ν(C=N) stretching vibrations arising from the terpy ligand at about 1590 cm^–1^ and those related to the presence of Pb–N bonds at 371 cm^–1^. An additional band associated with the ν(C≡N) stretching vibrations is observed at 2231 cm^–1^, indicating the presence of benzonitrile. In addition, an in-depth IR study at different temperatures shows the shift of both nitrile stretching vibrations to higher wavenumbers as the temperature decreases. The initial wavenumber vibrations of the nitrile group are recovered when returning to room temperature (RT) (Figure S4). A similar shift to higher wavenumbers when the benzonitrile molecule is coordinated has been observed in the literature with other transition metals^30^ and agrees with the observation in the X-ray diffraction studies at low and room temperatures. Similarly, the shift back to lower wavelengths when the temperature increases is because the percentage of benzonitrile molecules coordinated to the lead center decreases.

The matrix-assisted laser desorption ionization (MALDI)(−) spectrum of **1** shows the peak corresponding to the ion [Au(C_6_F_5_)_2_]^−^ (at *m*/*z* = 530) as a base peak (Figure S6). Besides, a peak due to the fragment [{Au(C_6_F_5_)_2_}{Pb(terpy)}]^+^ is detected at *m*/*z* = 972 in its MALDI(+) mass spectrum (Figure S7).

Regarding the ^1^H NMR spectra of **1** in [*D*_6_]-dimethyl sulfoxide (DMSO) (Figure S8), in addition to the six signals corresponding to the terpyridine ligand in the 8.74–7.52 ppm range, its ^1^H NMR spectrum displays resonances due to the aromatic protons of the benzonitrile between 7.86 and 7.57 ppm. On the other hand, its ^19^F NMR spectrum (Figure S9) shows the typical pattern corresponding to C_6_F_5_ groups bonded to gold(I), namely, three resonances at −114.6, −161.4, and −162.8 ppm, due to the *ortho*, *para*, and *meta* fluorine atoms, respectively. These chemical shifts, very similar to those of the gold precursor NBu_4_[Au(C_6_F_5_)_2_], suggest the rupture of the metallophilic interactions in solution.

Moreover, the molar conductivity of complex **1** in acetone, 241 Ω^–1^ cm^2^ mol^–1^, indicates its conductive behavior in solution as a 2:1 electrolyte, suggesting a complete dissociation of the complex in its ionic counterparts and the absence of metallophilic interactions in solution.

### Crystal Structure at Different Pressure

The presence of cavities in the supramolecular structure of the Au(I)–Pb(II) precursor and its ability to host solvent molecules prompted us to carry out in-depth X-ray diffraction studies at different pressures in order to check the response of this type of material under pressure conditions. Dark green single crystals of **1** suitable for X-ray diffraction studies were obtained by slow evaporation of a solution of the compound in benzonitrile. The structural changes at different pressures in a range from 0 to 2.1 GPa were followed by single-crystal X-ray diffraction. The crystals were subjected to high pressures, which have been applied using a diamond anvil cell (DAC), in particular, the Merrill–Bassett design DAC,^31,32^ at ambient temperature.

Daphne oil 7575 was used as the pressure-transmitting medium. X-ray diffraction was measured at the Material Science Beamline at the Swiss Light Source (λ = 0.49471 Å).^33^

Complex **1** crystallizes in the monoclinic space group *Cc* in the whole range of pressures studied. Its crystal structure consists of trinuclear Au_2_Pb units in which a [Pb(terpy)]^2+^ cation is surrounded by two [Au(C_6_F_5_)_2_]^−^ anions, which are held together through Au(I)···Pb(II) interactions. Aurophilic interactions between adjacent trinuclear units afford the growth of the intermetallic chain, resulting in the formation of a one-dimensional polymer. The chelating terpyridine ligand is bonded to the lead center through its three nitrogen atoms with Pb–N bond distances ranging from 2.41(6) to 2.61(5) Å in the whole range of pressures studied. All of them fall within the usual range for Pb(II) complexes with terpyridine or terpyridine-type ligands.^34,35^ There is also a benzonitrile molecule per Au_2_Pb unit occupying voids in the net.

**Figure 1:**
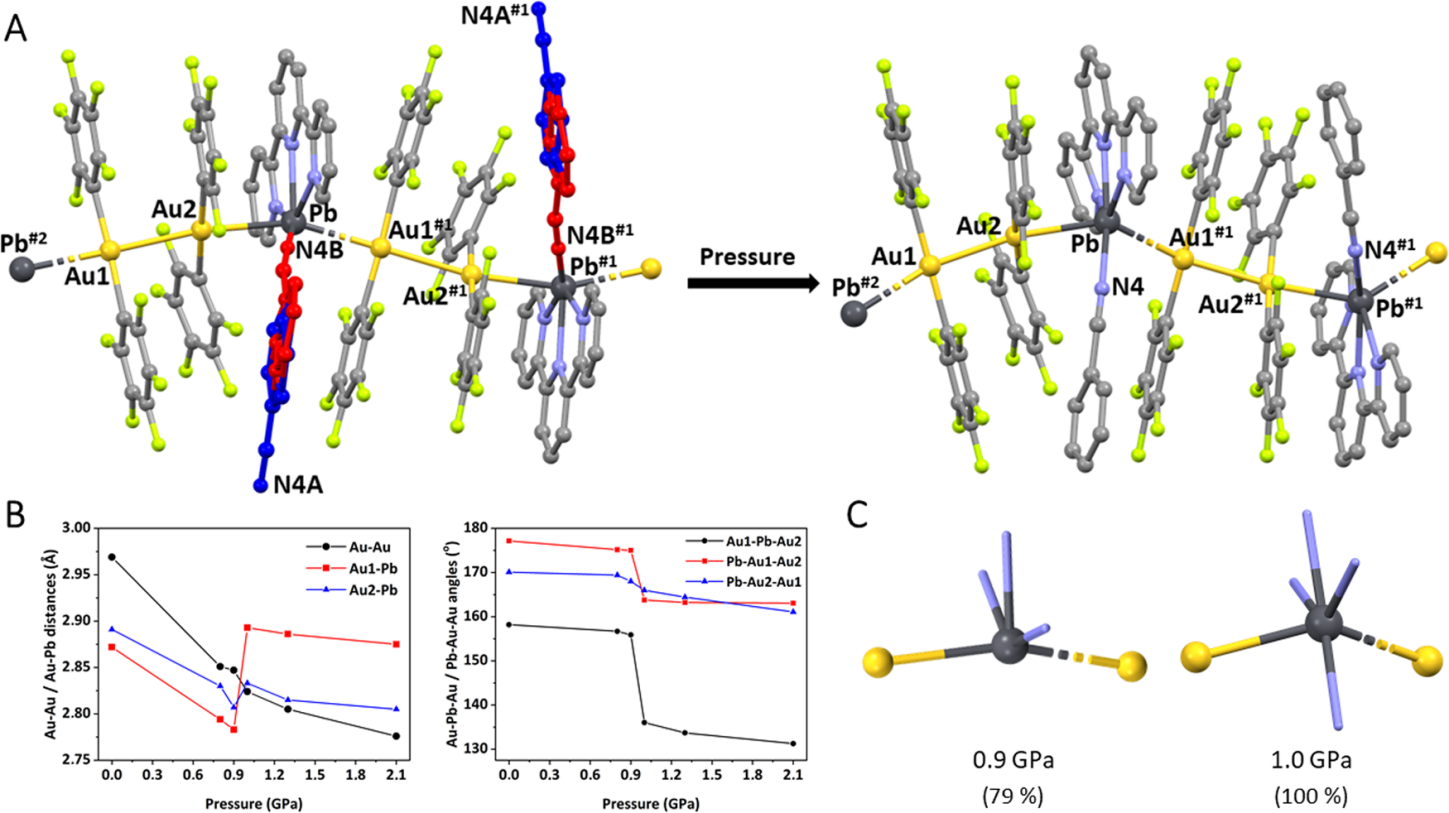
Pressure effect in the crystal structure of **1** at ambient pressure and at 2.1 GPa (A). All of the measurements were carried out at room temperature. Hydrogen atoms have been omitted for clarity. Red: benzonitrile coordinated, dark blue: benzonitrile not coordinated. #1 *x* + 1/2, −*y* + 3/2, *z* + 1/2; #2 *x* – 1/2, −*y* + 3/2, *z* – 1/2. Representation of the modification of Au–Au and Au–Pb distances (left) and Au–Pb–Au and Pb–Au–Au angles (right) *versus* pressure in **1** (B). Coordination environment of lead(II) in complex **1** at 0.9 for 79% of molecules (left) and 1.0 GPa for 100% of the molecules (right) (C).

At 0.1 MPa, the crystal is green and shows a Au–Au distance of 2.970(3) Å, but its color gets darker and the Au–Au distance decreases with increasing pressures, reaching a value as short as 2.776(3) Å at 2.1 GPa (Table 1 and Figure 1). Over the total pressure range studied, the Au–Au distance decreases a 6.5%.

**Table 1:** Intermetallic Distances and Angles at Different Pressures in **1**

*P* (GPa)	Au–Au (Å)	Au1–Pb (Å)	Au2–Pb (Å)	Au1–Pb–Au2 (deg)	Pb–Au1–Au2 (deg)	Pb–Au2–Au1 (deg)
Compression						
0.0	2.970(3)	2.871(3)	2.892(2)	158.2(2)	177.16(13)	170.2(2)
0.8	2.850(4)	2.795(3)	2.829(3)	156.8(4)	175.20(10)	169.4(4)
0.9	2.843(6)	2.791(8)	2.806(4)	155.6(5)	174.8(2)	168.2(5)
1.0	2.824(4)	2.893(4)	2.832(3)	136.03(18)	163.78(16)	165.99(13)
1.3	2.805(3)	2.886(3)	2.815(2)	133.71(14)	163.22(12)	164.43(10)
2.1	2.776(3)	2.875(3)	2.805(2)	131.29(14)	163.09(12)	161.07(10)
Decompression						
0.0	2.9693(16)	2.8607(17)	2.8989(14)	157.41(6)	177.21(6)	171.05(6)

In contrast, the Au···Pb interactions do not display the same behavior. Thus, although they suffer an initial contraction with increasing pressures (from 0.0 to 0.9 GPa), the Au–Pb distances increase between 0.9 and 1.0 GPa, decreasing again as the pressure increases from 1.0 to 2.1 GPa (Table 1 and Figure 1). Both Au–Pb distances follow the same trend, but they suffer a very different lengthening from 0.9 to 1.0 GPa, 3.8% in Au1–Pb and only 0.9% in Au2–Pb.

This different behavior of both Au–Pb distances with pressure leads to the fact that, over the total range of pressures studied, while the Au1–Pb distance hardly changes [2.871(3) Å at ambient pressure and 2.875(3) Å at 2.1 GPa], the Au2–Pb distance decreases by approximately 0.09 Å [from 2.892(2) Å at ambient pressure to 2.805(2) Å at 2.1 GPa]. Thus, the Au1–Pb distance increases by almost 0.1 Å, while the Au2–Pb distance only increases by about 0.03 Å.

It is worth noting that, as can be observed in Table 1 and Figure 1, and in spite of the smaller radius of gold, in the first range of pressures, the Au–Pb distances [2.871(3) and 2.892(2) Å at ambient pressure and 2.791(8) and 2.806(4) Å at 0.9 GPa] are shorter than the Au–Au one [2.970(3) Å at ambient pressure and 2.843(6) Å at 0.9 GPa]. Moreover, the Au–Pb distances at 0.9 GPa are even shorter than the sum of covalent radii of gold and lead (*r*_cov,Au_ = 1.36 Å, *r*_cov,Pb_ = 1.46 Å)^36^ and represent the shortest Au–Pb distances described so far.^29,37−41^

Nevertheless, the most dramatic and striking change that can be observed from 0.9 to 1.0 GPa involves the benzonitrile solvent molecule, which at ambient pressure is disordered between a nonconnected and a bonded position (Figure 1A, left), while at 1.0 GPa, it orders toward the Pb atom and effectively coordinates to it through the nitrogen atom of the benzonitrile molecule (Figure 1A, right). At 2.1 GPa, it displays a Pb–N bond distance of 2.80(5) Å, which is longer than those corresponding to the terpyridine ligand. Similar examples of pressure-induced bond formation have already been reported in the literature,^11,42,43^ but such a reorientation and non-intramolecular bond formation, occurring without loss of symmetry, has not been previously described, to the best of our knowledge.

Therefore, the full coordination of the solvent molecule is likely to be responsible for the marked lengthening observed in the Au···Pb interactions and for the sudden sharpening of the Au–Pb–Au angle, as shown in Table 1 and Figure 1. Although the Au–Pb–Au angle diminishes with increasing pressures in the whole range of pressures studied, from the total decrease of about 17°, it diminishes about 13° between 0.9 and 1.0 GPa. The same effect with pressure is observed in the Au–Au–Pb angles, although the sharpening between 0.9 and 1.0 GPa is not so noticeable in this case.

On the other hand, although the symmetry in the crystal does not vary upon increasing pressure and no change in the space group is detected, modifications in the external pressure affect the unit cell dimensions and volume (Table S1 and Figure S12). Thus, while the volume undergoes an almost regular contraction as the pressure increases, the β angle increases slightly from ambient pressure to 2.1 GPa, but more significantly between 0.9 and 1.0 GPa, as the benzonitrile molecule spins and binds the lead atom. Regarding the unit cell axes *a*, *b*, and *c*, in general, they all suffer a reduction with increasing pressures. However, between 0.9 and 1.0 GPa, the reduction in *a* is more significant than in the rest of the steps, while *b* and *c* increase instead of decreasing, lengthening that is more noticeable in *b* than in *c*. An analysis of principal compression axes using PASCal^44^ is reported in the SI (Tables S2 and S3 and Figures S13 and S14). Interestingly, it should be noted that the pressure effect is reversible after decompression, since if we reverse the pressure effect, the unit cell values as well as the values of distances and bond angles return to practically the same as at ambient pressure.

Finally, it is worth noting the differences observed in the coordination environment of lead in both complexes, as well as the effect of pressure in both cases. Thus, as shown in Figure 1C, the lead atom in **1** displays an hemidirected environment with a partial void for the lone electron pair of Pb(II).^45^ Besides, the full coordination of the benzonitrile molecule between 0.9 and 1.0 GPa provokes an increase in the nominal coordination number of lead from five to six and with the mentioned distortion of the Au–Pb–Au angle (from 155.6(5)° at 0.9 GPa to 136.03(18)° at 1.0 GPa).

### Crystal Structure at Different Temperatures

To prove whether the temperature was also able to induce structural changes in the crystal structure of complex **1**, we also carried out a crystallographic analysis at different temperatures. Thus, a single crystal of **1** suitable for X-ray diffraction studies was mounted in inert oil on a MiteGen MicroMount and transferred to the cold nitrogen stream of a Bruker APEX-II CCD diffractometer, equipped with a low-temperature controller system, and the study was performed at 285, 200, and 100 K. With increasing pressure, no change of symmetry is observed with temperature either.

**Figure 2:**
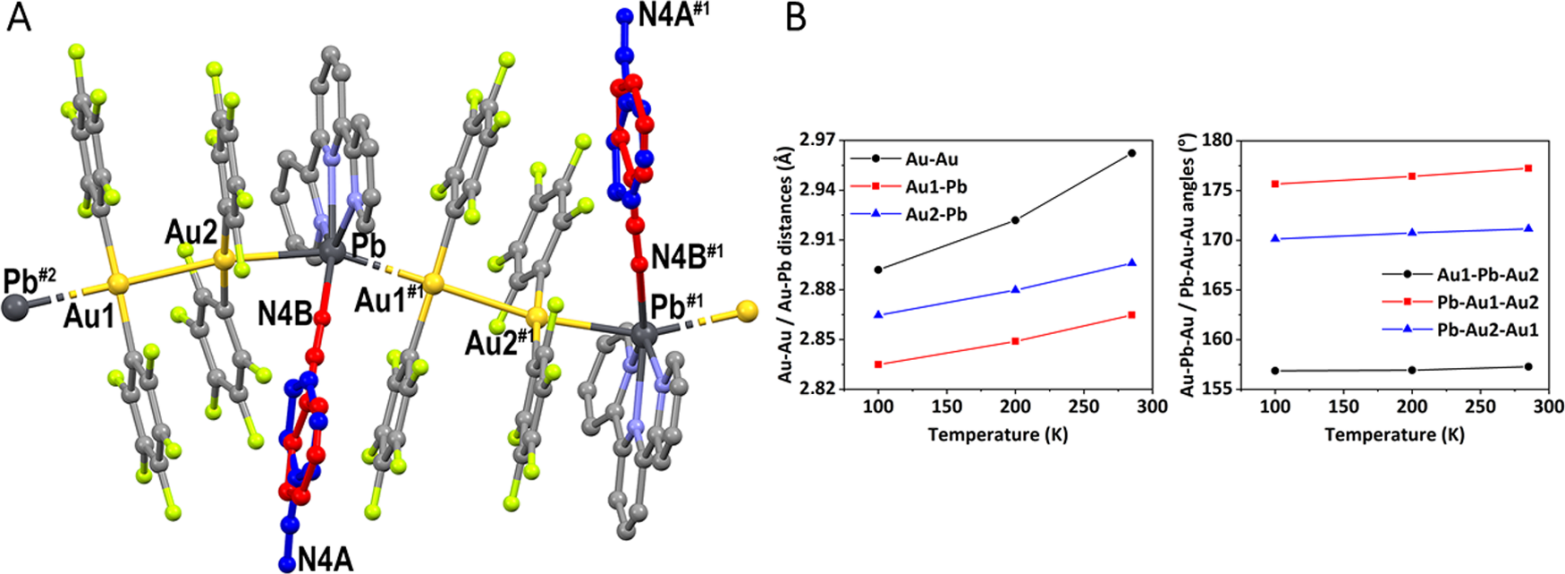
Crystal structure of **1** at room temperature showing the disordered in the benzonitrile molecule throughout the temperature range studied. Hydrogen atoms have been omitted for clarity (A). All of the measurements were carried out at ambient pressure. Red: benzonitrile coordinated, dark blue: benzonitrile not coordinated. #1 *x* – 1/2, −*y* + 1/2, *z* – 1/2; #2 *x* + 1/2, −*y* + 1/2, *z* + 1/2 (A). Representation of the modification of Au–Au and Au–Pb distances (left) and Au–Pb–Au and Pb–Au–Au angles (right) *versus* temperature in **1** (B).

As the temperature decreases, the crystal structure suffers a regular contraction that is evident in both the unit cell parameters and intermetallic distances and angles, which fits a linear dependence with temperature (Table S4 and Figure S15). Thus, the aurophilic interaction varies from 2.9623(7) Å at 285 K to 2.8920(5) Å at 100 K, while the Au–Pb distances also decrease regularly from 2.8648(7) and 2.8960(6) Å at 285 K to 2.8349(4) and 2.8646(4) Å at 100 K (Table 2 and Figure 2B).

**Table 2:** Intermetallic Distances and Angles at Different Temperatures in **1**

*T* (K)	Au–Au (Å)	Au1–Pb (Å)	Au2–Pb (Å)	Au1–Pb–Au2 (deg)	Pb–Au1–Au2 (deg)	Pb–Au2–Au1 (deg)
285	2.9623(7)	2.8648(7)	2.8960(6)	157.28(2)	177.24(2)	171.15(2)
200	2.9219(6)	2.8489(6)	2.8798(5)	156.93(2)	176.43(2)	170.74(2)
100	2.8920(5)	2.8349(4)	2.8646(4)	156.87(2)	175.66(1)	170.13(1)

However, while these parameters, as well as the orientation of the benzonitrile molecule, drastically change with increasing pressures from 0.9 to 1.0 GPa, the modifications in the structure as the temperature is lowered are gradual. Thus, the benzonitrile molecule also appears disordered in two different positions (uncoordinated–coordinated to lead) in the whole range of temperatures measured (Figure 2A), but the percentage of coordinated solvent increases as the temperature is lowered. The variation of these percentages with temperature also fits a straight line, showing values of 65–35% at 100 K, 70–30% at 200 K, and 74–26% at 285 K.

The extrapolation of these data leads to the conclusion that only by lowering the temperature, it is not possible to produce the total coordination of the solvent, since at 0 K, only 60% of the benzonitrile would be coordinated to the lead atom. On the contrary, the increase of the pressure produces a drastic change, thus acting as a switch for the coordination of the solvent molecule.

### Photophysical Properties

The commented pressure and temperature effects have a strong influence on the photophysical properties of complex **1**.

Thus, its UV–vis spectrum in the DMSO solution displays four maxima (Figure S16). The band at higher energy, located at 263 nm (ε = 2.8 × 10^4^ M^–1^ cm^–1^), appears at similar energy to that of the terpyridine ligand; therefore, it is likely to arise from π → π* transitions located in aromatic rings. The bands at 280 (ε = 2.2 × 10^4^ M^–1^ cm^–1^), 315 (ε = 9.7 × 10^3^ M^–1^ cm^–1^), and 335 nm (ε = 2.9 × 10^3^ M^–1^ cm^–1^) also appear in the spectrum of the gold precursor NBu_4_[Au(C_6_F_5_)_2_], and these can arise from internal π → π* transitions located in orbitals of the perhalophenyl groups and from charge-transfer transitions between Au(I) and π* orbitals. Nevertheless, the absence of bands at lower energies, usually observed in metal centered transitions, is indicative of the rupture of the intermetallic Au(I)···Pb(II) interactions in solution, as it was already suggested based on the molar conductivity measurements.

By contrast, the absorption spectrum of **1** in the solid state also exhibits absorption bands that can be related to transitions that appear in the precursors terpyridine (285 nm) and NBu_4_[Au(C_6_F_5_)_2_] (337 nm), as well as a band at 680 nm that do not appear in the absorption spectra of the precursors (Figure S17). Consequently, the origin of this new absorption could be associated with transitions involving the interacting metal centers. In fact, this absorption appears in the same energetic zone as that of the excitation spectrum at room temperature.

Also, compound **1** displays an intense luminescence in the solid state at room temperature and at 77 K (Figure S18) but, as it was expected, it is not luminescent in solution due to the rupture of the metal–metal interactions.

In the solid state at room temperature, complex **1** shows a dark red luminescence almost imperceptible to the human eye, since its emission band is centered at 780 nm (λ_exc_ = 600–750 nm, continuous excitation), at the limit of the visible region and with a photoluminescence quantum yield (PLQY) of 25%.

**Figure 3:**
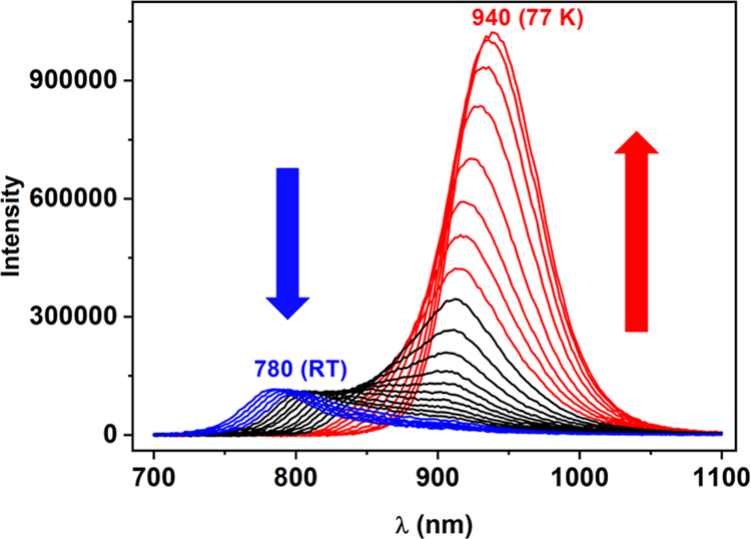
Superposition of emission spectra of complex **1** in the solid state from room temperature to 77 K every 10 K.

Very interestingly, at 77 K, a new emission band appears in the near-infrared (NIR) zone at 940 nm (λ_exc_ = 700–800 nm, continuous excitation). The shift of the emission to the red at low temperatures is common in complexes displaying unsupported intermetallic interactions when the metallic centers are involved in the transitions that give rise to the luminescence. This has been justified as a consequence of the thermal contraction of the structure at 77 K that reduces the metal–metal distances and, consequently, the highest occupied molecular orbital (HOMO)–lowest unoccupied molecular orbital (LUMO) gap.^46^ Nevertheless, in this case, the shift is too large (*ca*. 160 nm) compared to most of the examples described, which suggests additional changes in the structure and a new emissive low-energy state (Figure 3).

In addition, both bands show different Stokes shifts and lifetimes. The higher energy band displays a smaller Stokes shift and shorter lifetime (368 ns), while that at lower energy, which appears at 77 K, shows a higher Stokes shift and a more than 20-fold lifetime (7.522 μs). We can propose that different emitting states are responsible for both emissions, being likely fluorescence and phosphorescence processes, respectively.

Finally, in Figure 3, we can observe the luminescence spectra obtained when the temperature varies from room temperature to 77 K. It is evident that in that range, two different emitting species exist, losing intensity at higher energy and increasing in intensity at lower energy, when the temperature is lowered. In both emissions, a slight shift to the red is also observed when the temperature decreases. Obviously, if the responsible species for the emissions were a unique species, we should observe a progressive shift of the emission when the temperature changes. Therefore, we regard both forms of complex **1** as responsible for both emissions, that with the benzonitrile ligand interacting with lead at low temperatures and that in which this ligand does not interact at room temperature.

### Computational Studies

Density functional theory (DFT) and time-dependent DFT (TD-DFT) computational studies were carried out to confirm the origin of the photophysical properties displayed by complex **1**, when the benzonitrile solvent molecule binds to the lead(II) center at high pressures or low temperatures, and when this molecule does not interact with the metal center at RT and ambient pressure. The model systems **1a** and **1b** employed for these studies were taken from the X-ray diffraction structures obtained at ambient and at high pressures and were fully optimized at BP86/DFT level of theory (Figure S19). The models display hexanuclear units to represent the most important interactions found experimentally.

**Figure 4:**
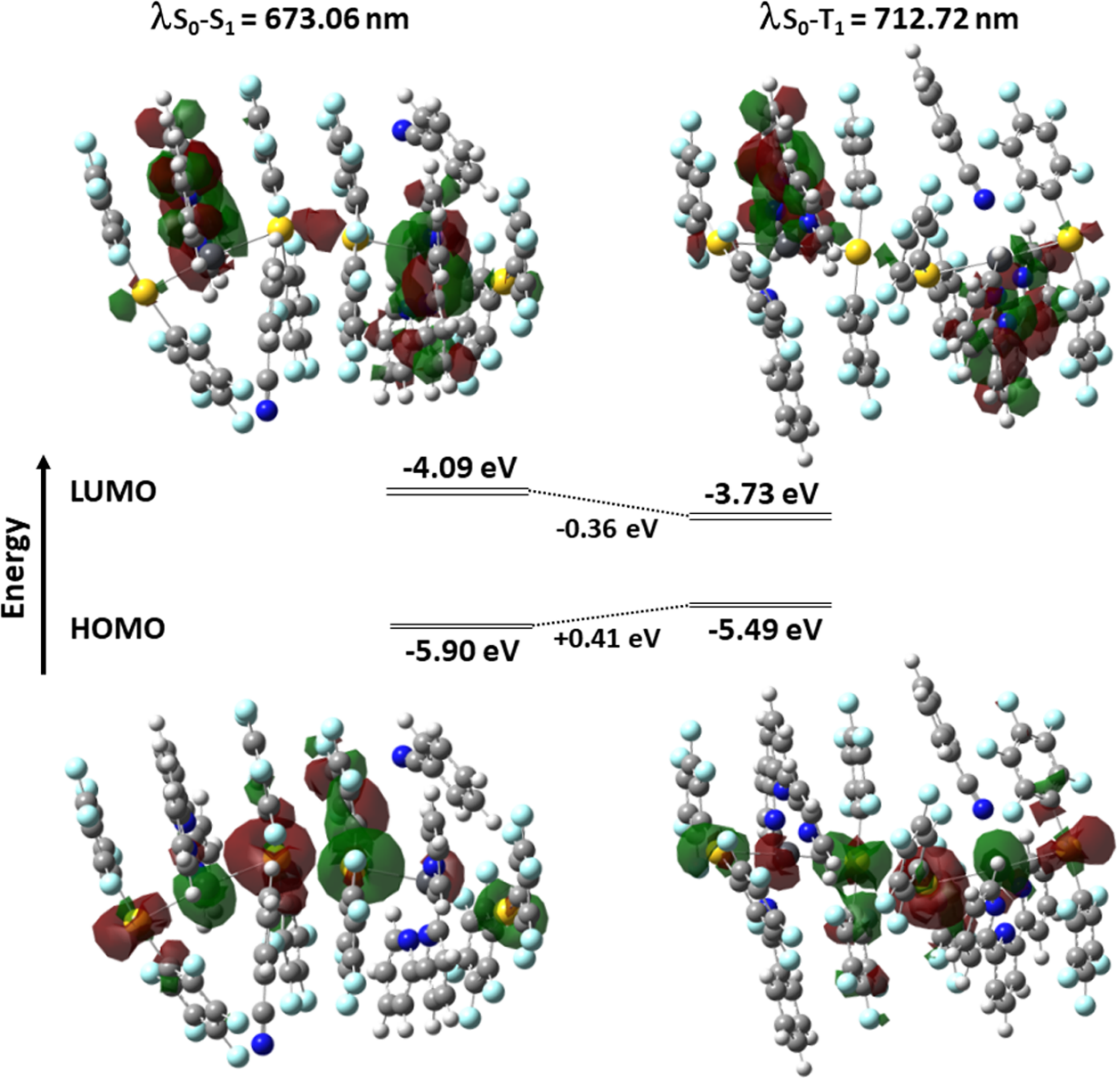
Computed TD-DFT S_0_ → S_1_ and S_0_ → T_1_ electronic excitations. Calculated energy diagrams for complex **1** with benzonitrile without interaction Pb–N (model **1a**, left) and with the benzonitrile oriented to the lead center (model **1b**, right).

The electronic structures of the model systems were computed through single-point DFT calculations. In both models, the HOMO is mostly located at the metal centers, largely at gold atoms, with a minor contribution from the perhalophenyl groups (Table S7). On the other hand, the LUMO is mainly placed at the terpy ligand with some contribution from the Pb(II) atoms, indicating that the terpy ligand has a very relevant role in the luminescence behavior of this complex. This character of the frontier MOs is likely to agree with metal-to-ligand charge-transfer (MLCT) transitions in both situations, in which the benzonitrile molecule changes its coordinative nature. The effect of the coordination of benzonitrile molecule to the lead centers lowers the LUMO level (−0.36 eV) relative to the one computed for the model system, in which the interaction is absent (−4.09 eV), whereas the HOMO level is slightly elevated (+0.41 eV), which results in the diminished HOMO–LUMO gap (Figure 4).

To confirm the origin of the electronic transition responsible for the luminescence emission of complex **1**, we performed an analysis of the energy of the lowest singlet–singlet and singlet–triplet electronic excitations for fluorescent model **1a** (at ambient pressure) and phosphorescent model **1b** (at high pressure), respectively, computed using the TD-DFT approach (Figure S22). In both cases, these lowest excitations correspond to the HOMO–LUMO transition. The predicted HOMO–LUMO transitions at 673 (noninteracting benzonitrile) and 713 nm (bind benzonitrile) agree with the previously assigned transition from the metal centers to the terpyridine ligand (MLCT) when the electronic structures were analyzed (*vide supra*). The computed excitations match the experimentally observed ones since as the temperature decreases or pressure increases, both the emission and excitation bands are red-shifted, giving rise to the species with the benzonitrile molecule oriented toward the Pb(II) center.

Moreover, there are two other high-intensity singlet–singlet excitations at 629 and 499 nm. The first one consists again of a HOMO → LUMO transition. The excitation at 499 nm consists of a HOMO → LUMO + 1 transition, in which the LUMO + 1 orbital is located on the terpy ligand and, therefore, it could be assigned to a charge transfer from the perhalophenyl ligands to the neutral ligand (LLCT).

**Figure 5:**
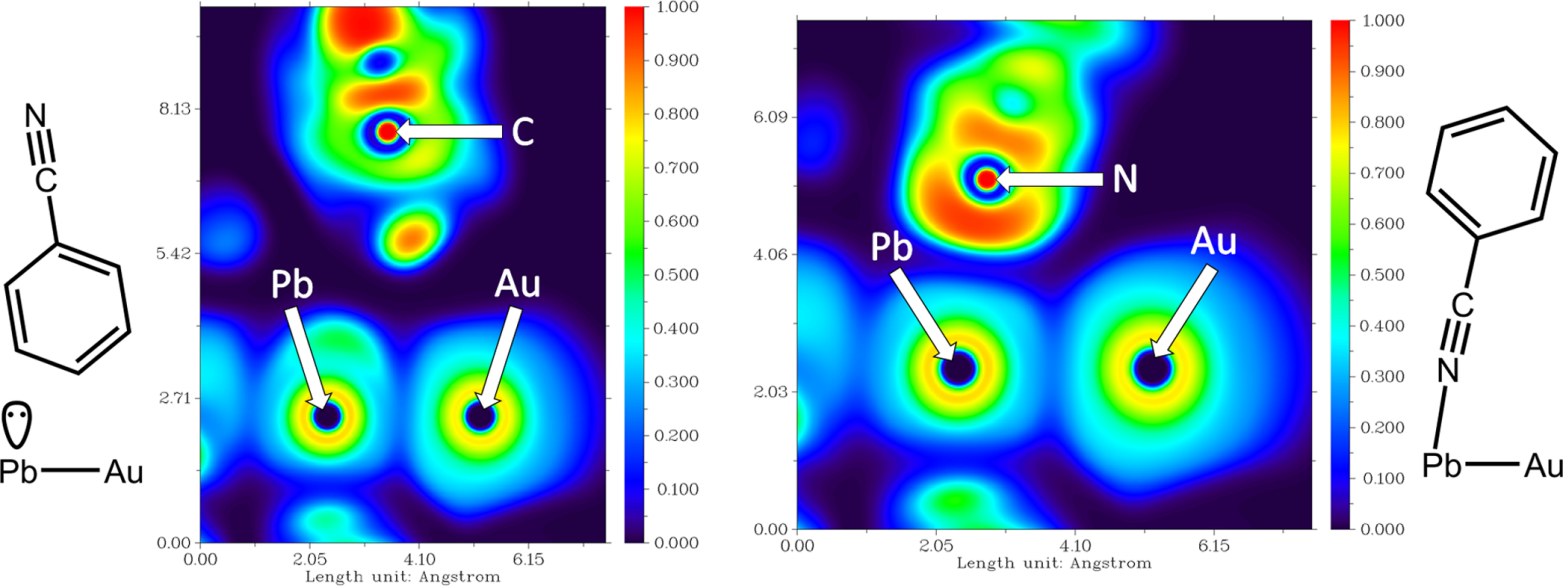
Two-dimensional (2D)-ELF plots for model systems **1a** (left) and **1b** (right).

A very interesting consequence of the benzonitrile reorientation at high pressure is the drastic change of the Pb(II) lone pair location when the benzonitrile ligand is bonded to Pb(II). To explain this, we have analyzed the electron localization function (ELF) for models **1a** and **1b**. Figure 5 depicts the Pb(II) coordination environments at ambient and at high pressures. When the benzonitrile ligand is only occupying a void but is not coordinated to Pb(II) in model **1a** (Figure 5, left), the lone pair occupies the vacant coordination site, but when the benzonitrile ligand is reoriented at high pressure and the nitrogen atom is bonded to the Pb(II) center, the lone pair disappears from the plane defined by the Au–Pb–N_benzonitrile_ sequence (Figure 5, right). Three-dimensional (3D) ELF plots (Figure S23) allow the location of the lone pair in model **1b** with a slightly lower probability (0.4 *versus* 0.5) than in model **1a**.

## Conclusions

Subjection of crystal of {[{Au(C_6_F_5_)_2_}_2_{Pb(terpy)}]·NCPh}*_n_* (**1**) to increasing pressures leads to a general and expected decrease of the metal–metal distances. Very interestingly, additional modifications of different nature in the crystal structure are also observed without undergoing a symmetry change. Surprisingly, the benzonitrile molecule in **1** experiences an unprecedented sudden reorientation and coordination to lead between 0.9 and 1.0 GPa. Furthermore, in this communication, the shortest Au(I)–Pb(II) distance reported in the literature so far is collected, being shorter than the sum of their covalent radii. On the other hand, the effect of temperature gives rise to a similar trend, although a complete reversal of the ligand arrangement is not possible. These effects have a very strong influence on the optical properties, leading to different emitting states whether the benzonitrile ligand is only occupying a void in the supramolecular structure or it is directly coordinated to lead. Computational studies support this different photophysical behavior and also account for the drastic change of the lead(II) lone pair upon benzonitrile coordination.

Further experiments on the ability of this starting complex **1** to incorporate different small molecules in liquid or gas phase are now under study.

## Experimental Section

### General

The starting product [{Au(C_6_F_5_)_2_}_2_{Pb(terpy)}]*_n_* was prepared according to the literature.^29^

### Materials and Physical Measurements

Infrared spectra were recorded in the range 4000–225 cm^–1^ on a Nicolet Nexos FT-IR Spectrum (Thermo Nicolet Corporation, Madison, WI, USA) using Nujol mulls between polyethylene sheets, andin the 4000–450 cm^–1^ range on a PerkinElmer FTIR Spectrum 1000 spectrophotometer. ^1^H and ^19^F NMR spectra were recorded on a Bruker Avance 300 in dimethyl sulfoxide solutions. Chemical shifts were quoted relative to SiMe_4_ (^1^H external) and CFCl_3_ (^19^F external). C, H, and N analyses were carried out with a C.E. Instrument EA-1110 CHNSO microanalyzer. The MALDI mass spectra were registered on a Microflex Bruker spectrometer using dithranol (DIT) and *trans*-2-(3-(4-*tert*-butylphenyl)-2-methyl-2-propenylidene)-malononitrile (DCTB) as the matrix. The *m*/*z* values are given for the higher peak in the isotopic pattern. Excitation and emission spectra in the solid state were recorded with an Edinburgh FLS 1000 fluorescence spectrometer. Luminescence lifetime was measured on an Edinburgh FLS 1000 fluorescence spectrometer. Quantum yields were measured in the solid state using a Hamamatsu Quantaurus-QY C11347-11 integrating sphere with excitation at 700 nm.

### Synthesis of {[{Au(C_6_F_5_)_2_}_2_{Pb(terpy)}]·NCPh}*_n_* (**1**)

A solution of [{Au(C_6_F_5_)_2_}_2_{Pb(terpy)}]*_n_* (0.088 g, 0.4 mmol) in benzonitrile (10 mL) was stirred for 10 min. Evaporation of the solvent to dryness gave rise to a dark green solid in an almost quantitative yield. ^1^H NMR (300 MHz, [*D*_6_]-DMSO, 298 K), δ 8.74 (m, 2H, H_1_), 8.64 (m, 2H, H_4_), 8.46 (d, 2H, H_5_, ^3^*J*(H_5_–H_6_) = 7.84 Hz), 8.13 (m, 1H, H_6_), 8.03 (td, 2H, H_3_, ^3^*J*(H_3_–H_2_) ∼ ^3^*J*(H_3_–H_4_) = 7.67 Hz, ^4^*J*(H_3_–H_1_) = 1.74 Hz), 7.52 (m, 2H, H_2_), 7.86-7.57 (m, 5H, CH) ppm. ^19^F NMR (282 MHz, [*D*_6_]-DMSO, 298 K) δ −114.58 (m, 4F, F_*o*_), −161.43 (t, 2F, F_*p*_, ^3^*J*(F_*p*_–F_*m*_) = 21.2 Hz), −162.78 (m, 4F, F_*m*_) ppm. FTIR (Nujol mulls): ν = 770, 955, 1504 cm^–1^ (Au–C_6_F_5_), ν = 1590 cm^–1^ (C=N), ν = 2231 cm^–1^ (C≡N), ν = 371 cm^–1^ (Pb–N). MALDI(+): *m*/*z* (%): 972 (100) [Au(C_6_F_5_)_2_Pb(terpy)]^+^; MALDI(−): *m*/*z* (%): 530 (100) [Au(C_6_F_5_)_2_]^−^; elemental analysis calcd (%) for C_46_H_16_Au_2_F_20_N_4_Pb: C, 34.41; H, 1.00; N, 3.49. Found: C, 34.06; H, 1.30; N, 3.75. Λ_M_ (acetone): 241 Ω^–1^ cm^2^ mol^–1^.

### Crystallography at Different Temperatures

The single-crystal X-ray diffraction data for **1** at ambient pressure were mounted in inert oil on a MiteGen MicroMount and transferred to the cold gas stream of a Bruker APEX-II CCD diffractometer equipped with an Oxford Instruments low-temperature attachment. Data were collected using monochromated Mo Kα radiation (λ = 0.71073 Å). Scan type: ω and ϕ. Absorption corrections: semiempirical (based on multiple scans). The structures were solved with the XT structure solution program using intrinsic phasing, refined with the SHELXL refinement package using least squares minimization, and refined on *F*_0_^2^ using the program SHELXL-97. Hydrogen atoms were included using a riding model. CCDC 2127710–2127712 contains the supporting crystallographic data for this paper.

### Synchrotron Single-Crystal X-ray Diffraction Experiments (Different Pressures)

Single crystals of the studied compounds were loaded in a Merrill–Basset diamond anvil cell (DAC)^31^ with 0.5 mm diamond culets. Crystals were placed inside preindented steel gaskets with a drilled 250 μm sample chamber. The pressure was calibrated in all experiments by ruby fluorescence.^47^ The ruby crystals were placed in two positions around the single crystal in order to better detect any significant pressure gradients appearing above the hydrostatic limit of the pressure-transmitting medium. X-ray diffraction experiments were carried out at the Materials Science Beamline at the Swiss Light Source.^33^ The CrysAlisPro^48^ program suite was used for the determination of the orientation matrices and initial data reduction. Structures were refined with SHELXL incorporated in Olex2.^49^ CCDC 2163070–2163075 contains the supporting crystallographic data for this paper.

### Computational Details

All calculations were performed using the Gaussian 16 suite of programs.^50^ We have performed single-point calculations on all model systems at the DFT-D3/BP86 level,^51^ including the empirical dispersion correction by Grimme *et al*.^52^ This level of theory has been proven to represent noncovalent interactions at lower computational cost. For these calculations, the corresponding def2-TZVP basis sets were used.^53^ The convergence criterion used for DFT calculations is 1 × 10^–6^.

## Supporting Information Available

Experimental and theoretical characterization of the complexes (PDF)
