# Supplementary material for: Switching On/Off of a Solvent Coordination in a Au(I)–Pb(II) Complex: High Pressure and Temperature as External Stimuli
Source: Inorg Chem. 2023 Jun 16;62(26):10307–16. doi: 10.1021/acs.inorgchem.3c01130 (PMC10862548; doi:10.1021/acs.inorgchem.3c01130)
Supplement: Supplementary file 1 — ic3c01130_si_001.pdf [file ic3c01130_si_001.pdf]

SUPPORTING INFORMATION

Switching on/off of a solvent coordination in a Au(I)-Pb(II) complex: high-pressure and temperature as external stimuli

Sonia Moreno,[a] Nicola Casati,[b]* María Rodríguez-Castillo,[a] Miguel Monge,[a] M. Elena Olmos,[a]* and José M. López-de-Luzuriaga[a]*

[a]Departamento de Química, Universidad de La Rioja, Centro de Investigación en Síntesis Química (CISQ), Complejo Científico-Tecnológico, 26006 – Logroño, Spain.

[b]Laboratory for Synchrotron Radtiation – Condensed Matter, Paul Scherrer Institute (PSI), WLGA/229 Forschungsstrasse 111, 5232 Villigen, Switzerland.

Table of Contents

I. Characterization of the complexes 2

1. IR spectra...3

2. Mass spectrometry data……………………………………………………………5

3. ^{1}H NMR spectra (300 MHz, 298K) 6

4. ^{19}F NMR spectra (282 MHz, 298K) 7

5. Single crystal analysis of compound **1**...8

II. Optical properties 18

1. UV-Vis absorption spectra in solution 18

2. UV-Vis absorption spectra in solid state 18

3. Photophysical properties 19

III. Computational studies 19

1. Orbitals involved in most important transitions for model **1a**……..................................20

2. Orbitals involved in most important transitions for model **1b**……..................................20

3. 3-D Electron Localization Function (ELF) for models **1a** and **1b**..................................22

I. Characterization of the complexes

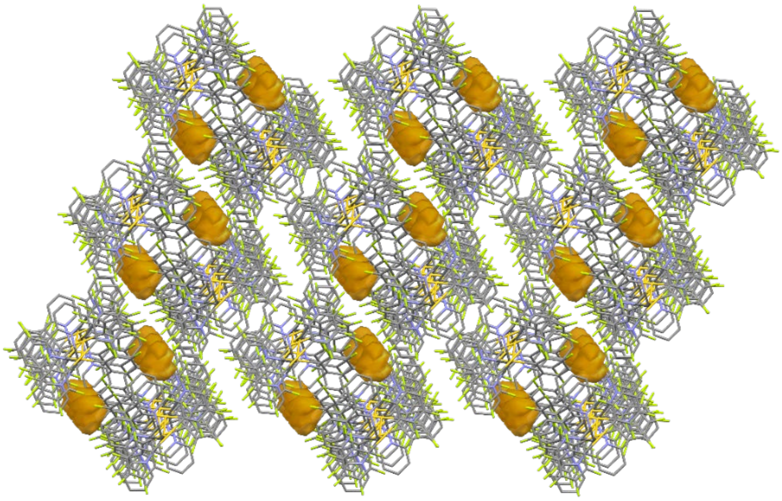

Figure S1. Crystal structure of compound $[\{Au(C_6F_5)_2\}_2\{Pb(terpy)\}]_n$ as seen through the crystallographic *b-axis*, where the orange spheres represent the channels.

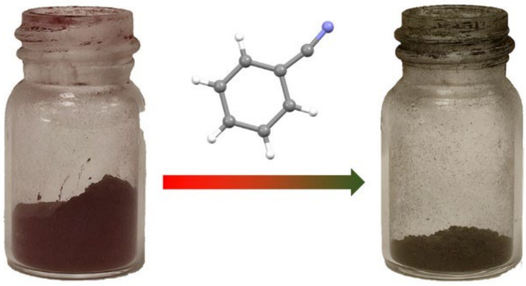

Figure S2. Colour of the solid $[\{Au(C_6F_5)_2\}_2\{Pb(terpy)\}]_n$ (left) and of the solid when it is exposed to benzonitrile (right, complex **1**).

1. IR SPECTRA

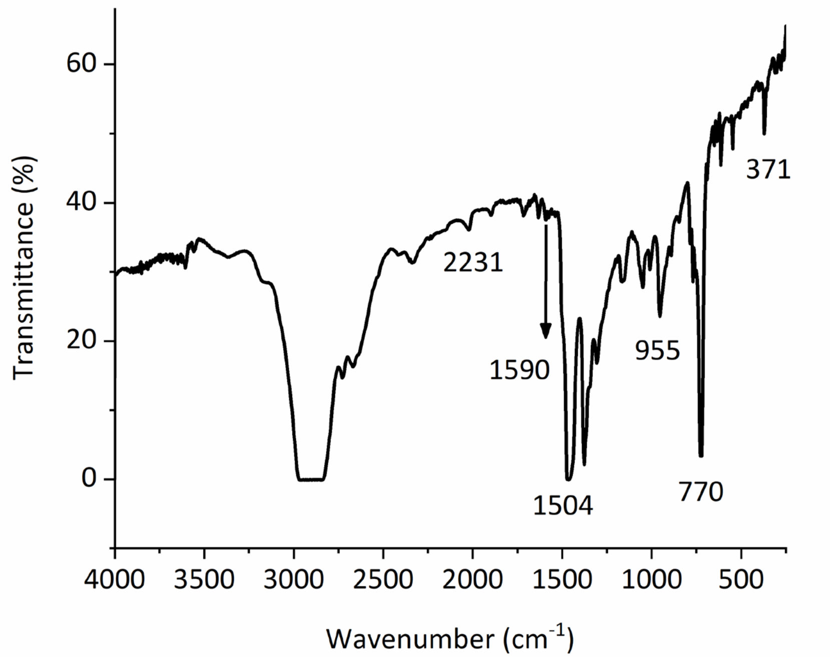

Figure S3. FT-IR spectrum of complex **1**.

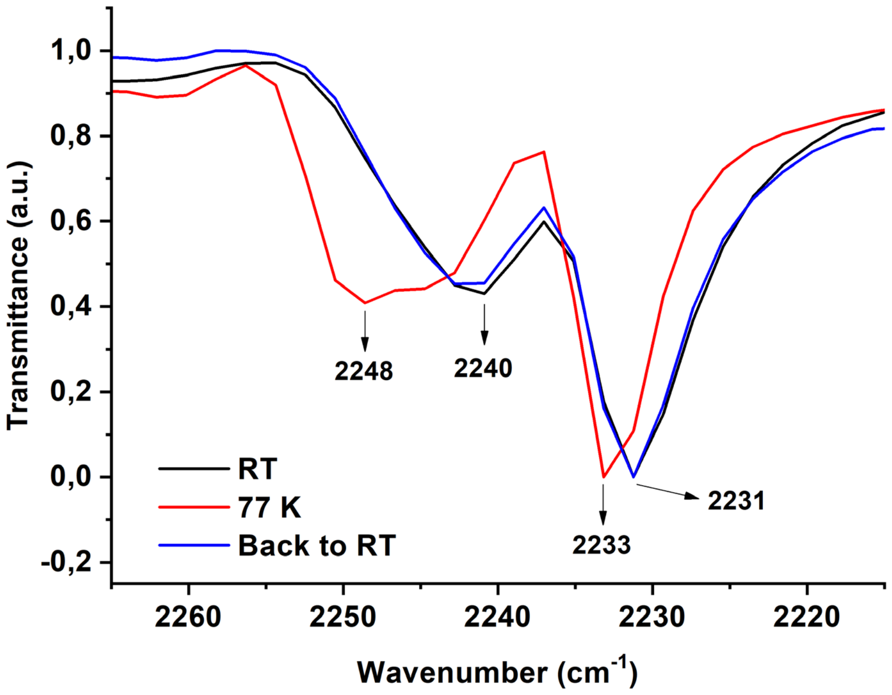

Figure S4. FT-IR spectrum of complex **1** at RT (black), 77 K (red) and back to room temperature from 77 K (blue).

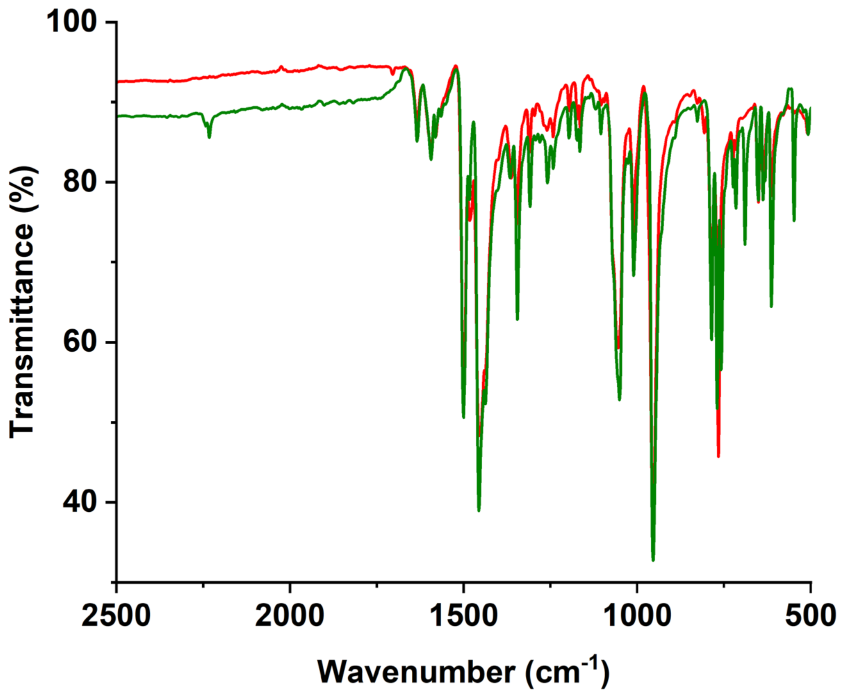

Figure S5. FT-IR spectra of complex **1** (green) and of Au-Pb precursor (red).

2. MASS SPECTROMETRY DATA

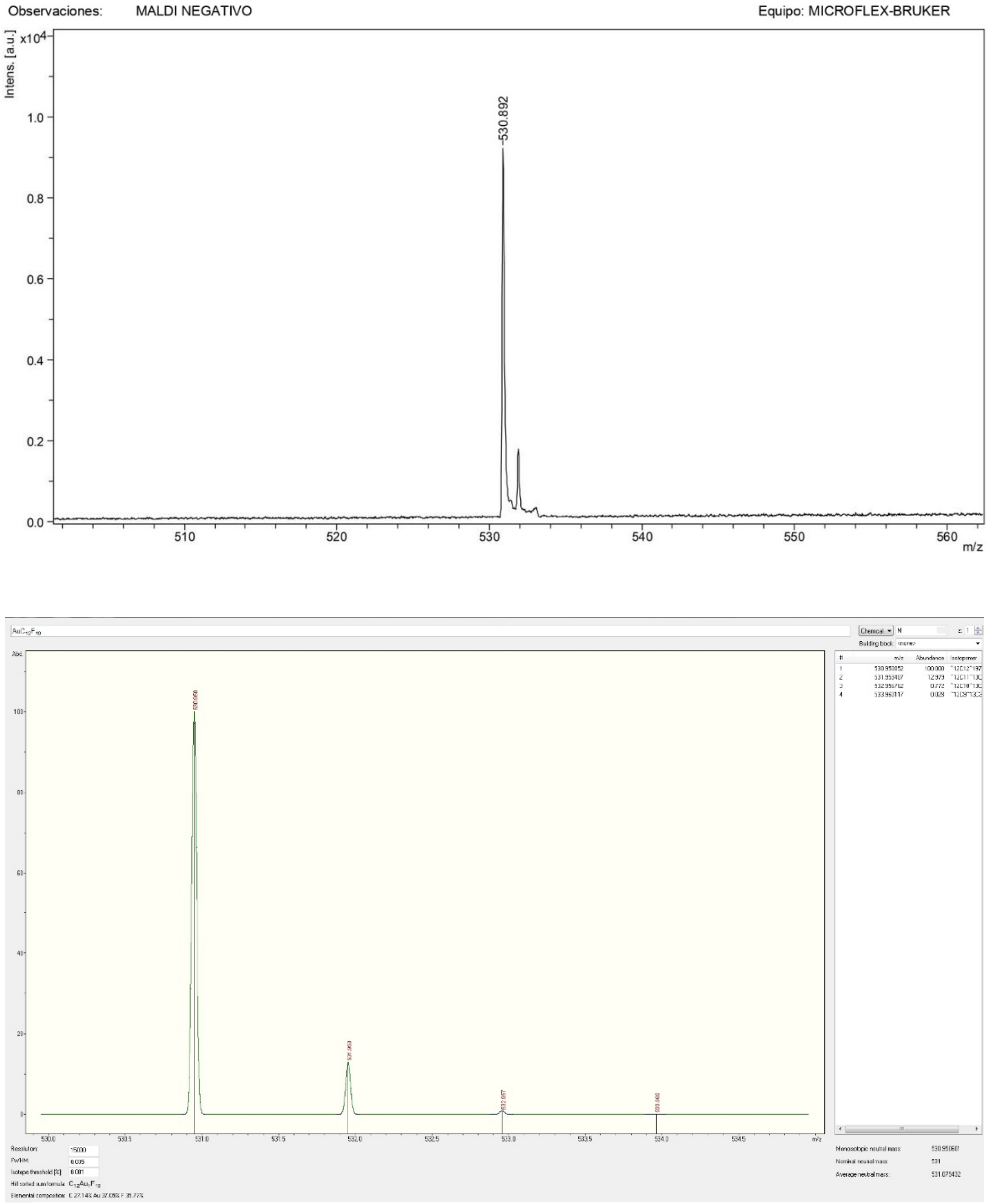

Figure S6. Spectrum of experimental (top) *versus* theoretical (bottom) negative mass spectrometry data.

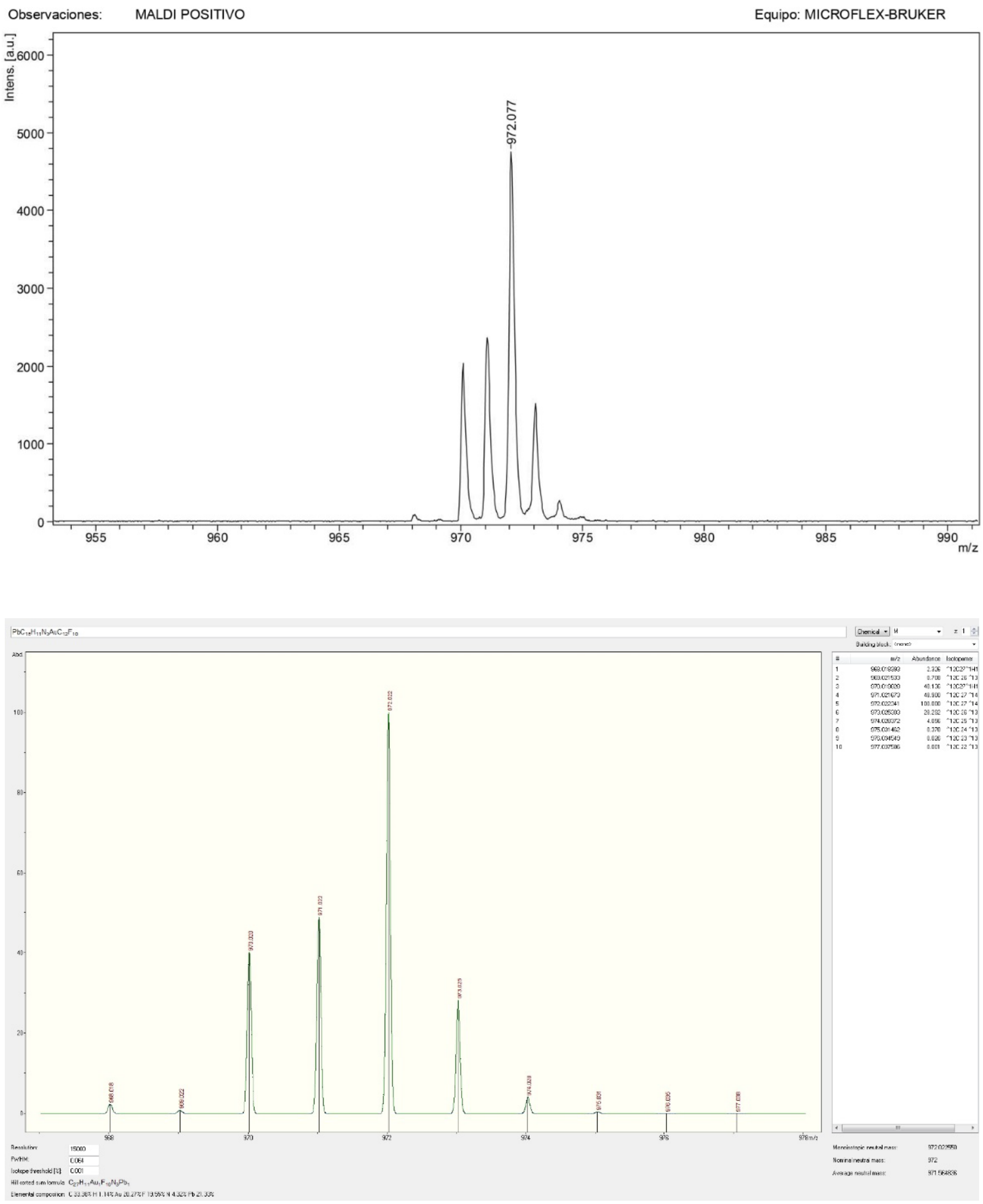

Figure S7. Spectrum of experimental (top) *versus* theoretical (bottom) positive mass spectrometry data.

3. ^{1}H NMR SPECTRA (300 MHZ, 298K)

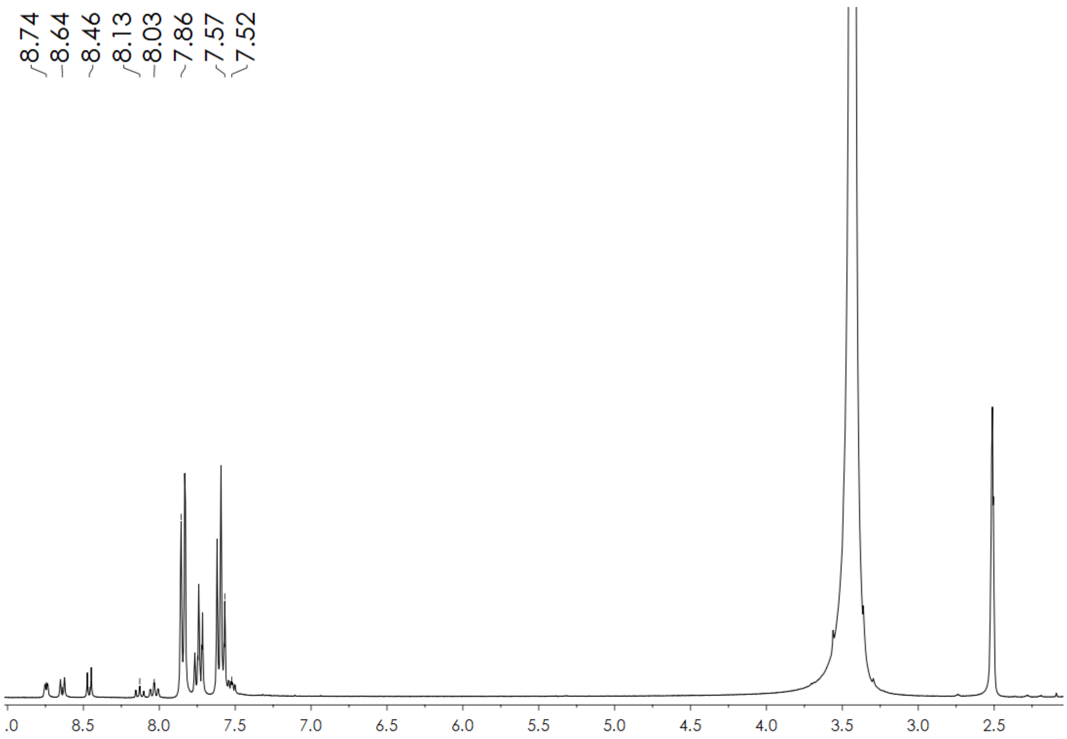

Figure S8. ^{1}H NMR spectrum of complex **1** in [D$_6$]-DMSO.

4. ^{19}F NMR SPECTRA (282 MHZ, 298K)

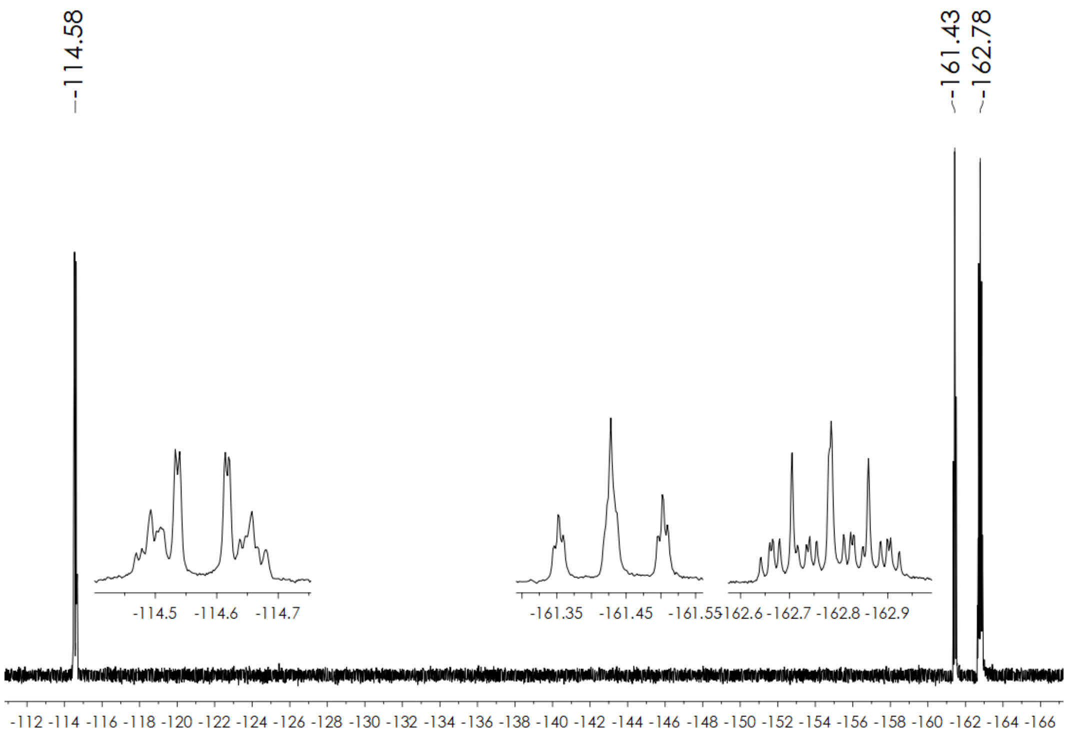

Figure S9. ^{19}F NMR spectrum of complex **1** in [D$_6$]-DMSO.

5. SINGLE CRYSTAL ANALYSIS OF COMPOUND 1

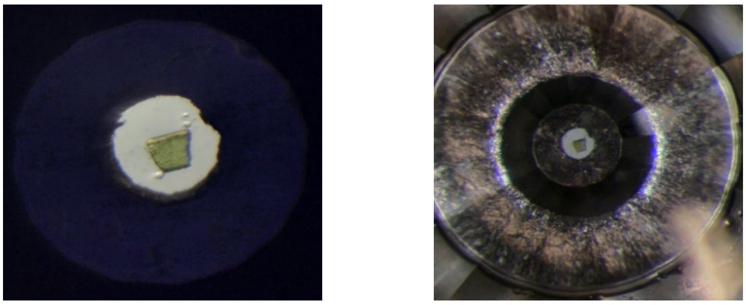

Figure S10. Single crystal of **1** placed on the diamond culet alongside small ruby spheres (left) and in the closed DAC at 0.0 GPa (right).

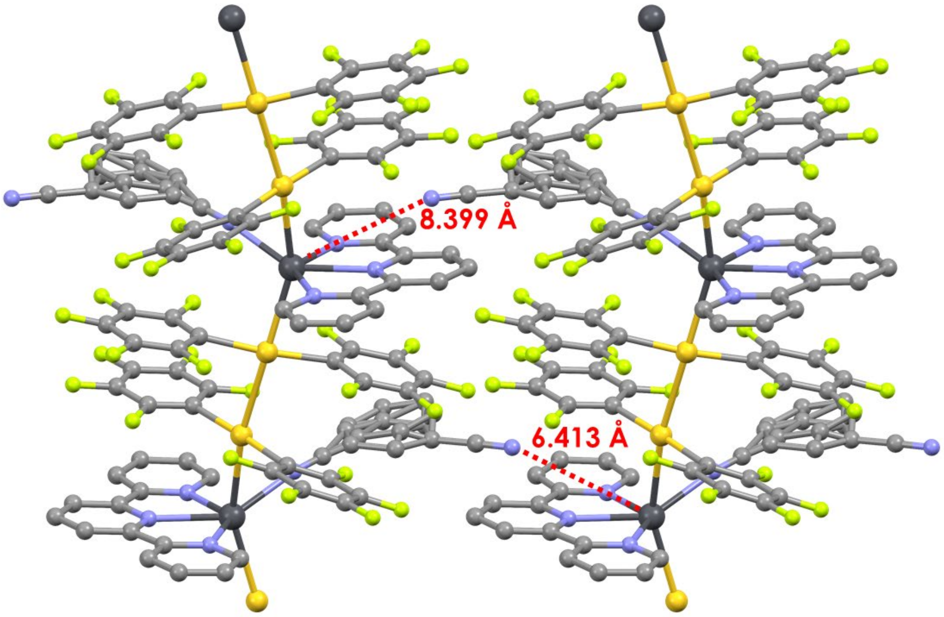

Figure S11. Two adjacent chains of crystal structure of complex **1** from *a* axis.

Table S1. Unit cell parameters at different pressures in **1**.

P (GPa)	a (Å)	b (Å)	c (Å)	β (°)	V (Å^3)
			**Compression**		
**0.0**	11.6663(4)	25.344(9)	15.8702(7)	105.282(3)	4526.4(16)
**0.8**	11.4740(6)	24.883(13)	15.3066(9)	105.612(5)	4209(2)
**0.9**	11.3931(12)	24.85(2)	15.2236(17)	105.695(11)	4150(3)
**1.0**	10.9626(4)	25.416(7)	15.3273(5)	107.416(4)	4078.8(12)
**1.3**	10.8530(3)	25.557(8)	15.1439(6)	107.531(3)	4005.4(12)
**2.1**	10.7511(5)	25.377(9)	14.9000(6)	107.645(5)	3873.9(13)
			**Decompression**		
**0.0**	11.6611(4)	25.3744(11)	15.8872(5)	105.402(3)	4532.1(3)

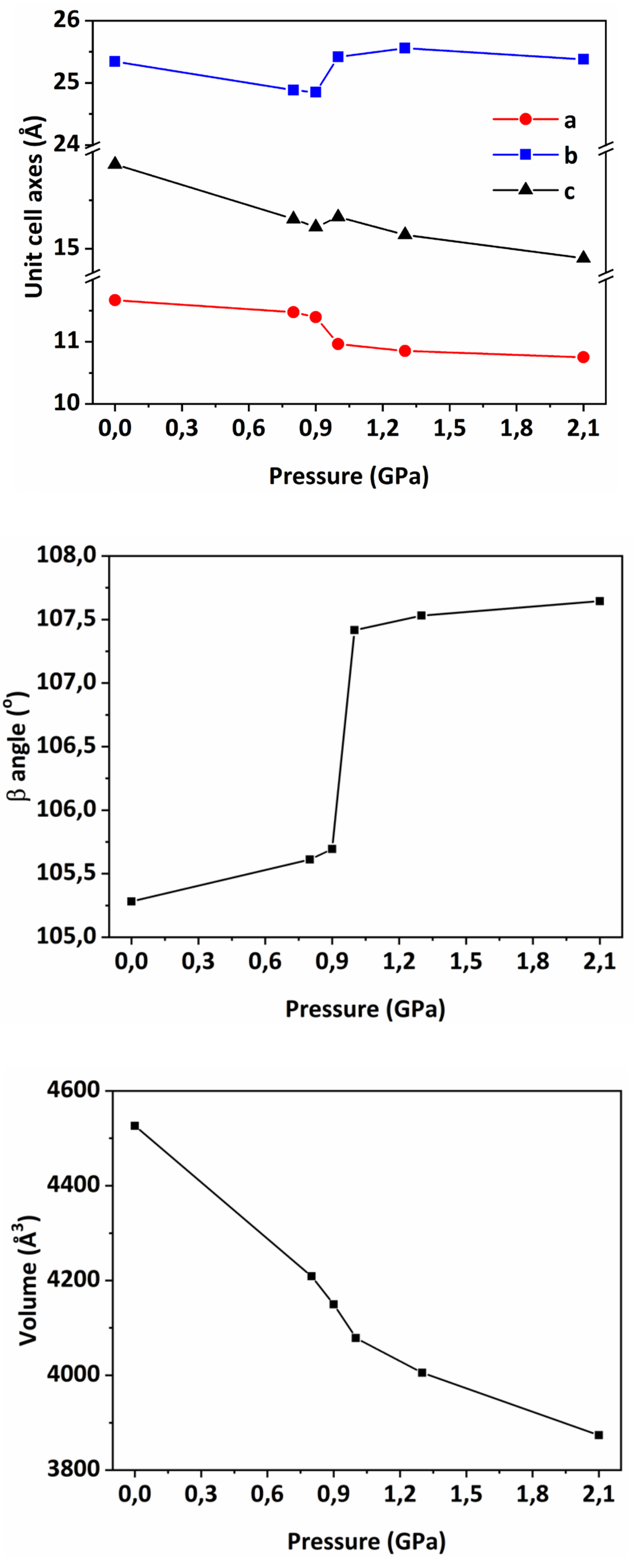

Figure S12. Representation of the modification of *a*, *b* and *c* axes (top), β angle (centre), and unit cell volume (bottom) *vs* pressure in **1**.

Table S2. Principal coefficients of thermal expansion and corresponding relative directions at different pressures in **1**.

Axis n	α_n (TPa^{-1})	Component along *a*	Component along *b*	Component along *c*	Approximate axis
**1**	44(23)	-0.9138	0.0000	-0.4061	[-2 0 -1]
**2**	27(6)	0.4841	0.0000	-0.8750	[1 0 -2]
**3**	-4.3(0)	0.0000	1.0000	0.0000	[0 1 0]

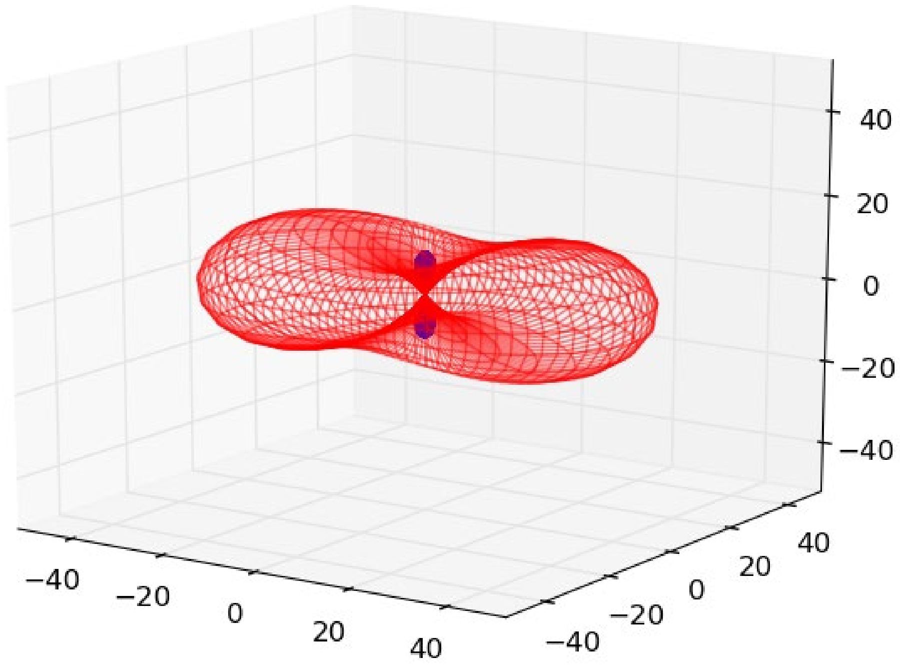

Figure S13. Thermal expansivity indicatrix at different pressures in **1**.

Table S3. Principal coefficients of thermal expansion and corresponding relative directions at different temperatures in **1**.

Axis n	α_n (MK^{-1})	Component along *a*	Component along *b*	Component along *c*	Approximate axis
**1**	10.2(6)	-0.9961	0.0000	0.0878	[-1 0 0]
**2**	40(2)	0.0000	1.0000	-0.0000	[0 1 0]
**3**	107(4)	0.4564	-0.0000	0.8898	[1 0 2]

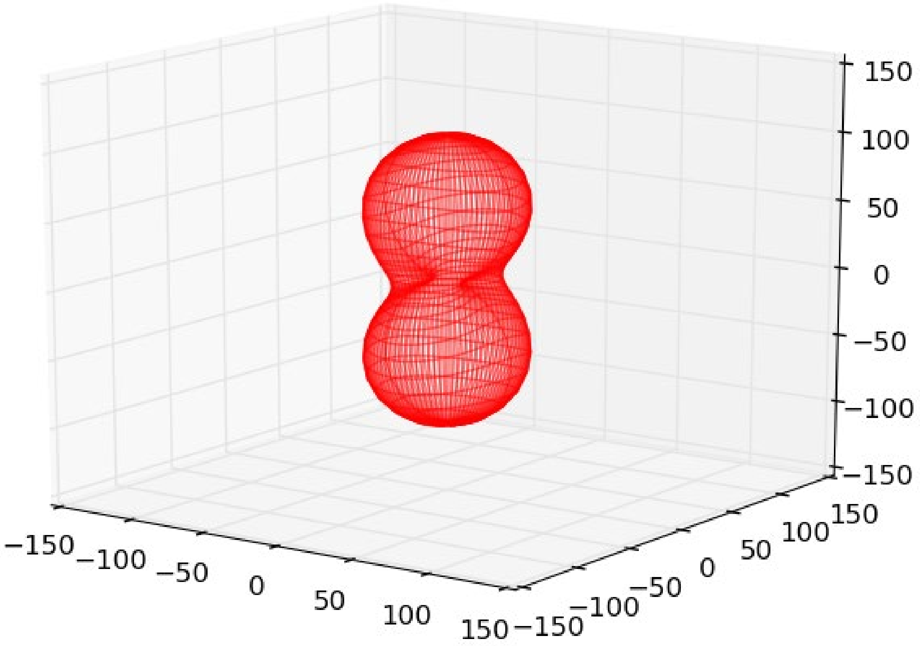

Figure S14. Thermal expansivity indicatrix at different temperatures in **1**.

Table S4. Unit cell parameters at different temperatures in **1**.

**T (K)**	**a (Å)**	**b (Å)**	**c (Å)**	**□ (°)**	**V (Å^3)**
**285**	11.6283(7)	25.3116(15)	15.8823(8)	105.370(2)	4507.5(4)
**200**	11.6160(6)	25.2465(14)	15.7504(8)	105.727(2)	4446.1(4)
**100**	11.6003(8)	25.1273(17)	15.6246(9)	105.933(2)	4379.4(5)

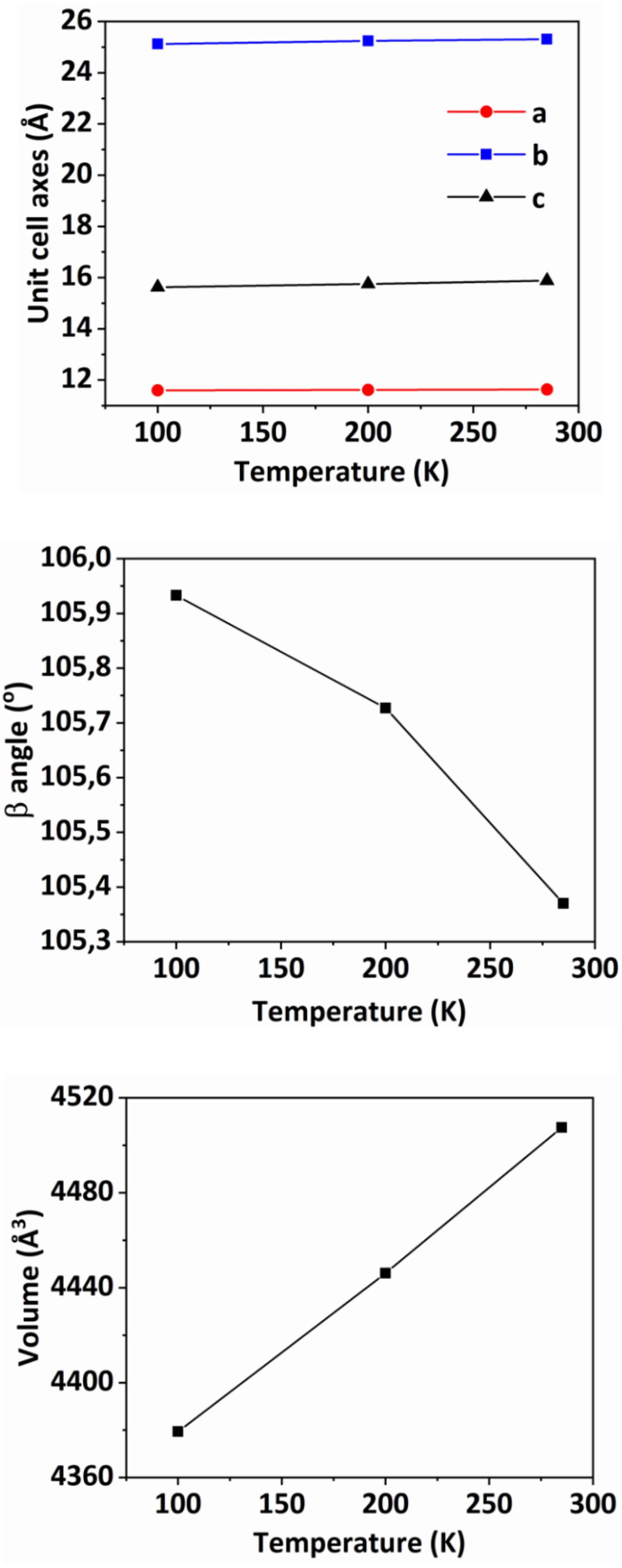

Figure S15. Representation of the modification of *a*, *b* and *c* axes (top, right), β angle (centre), and unit cell volume (bottom) *vs* temperature in **1**.

Table S5. Crystallographic data for **1** between ambient pressure and 2.1 GPa at ambient temperature.

	**0 GPa**	**0.8 GPa**
Chemical Formula	$C_{46}H_{16}PbAu_2F_{20}N_4$	$C_{46}H_{16}PbAu_2F_{20}N_4$
Crystal habit	Green prism	Green prism
Crystal size/mm	0.06x0.06x0.025	0.06x0.06x0.025
Crystal system	Monoclinic	Monoclinic
Space group	*Cc*	*Cc*
a/Å	11.6663(4)	11.4740(6)
b/Å	25.344(9)	24.883(13)
c/Å	15.8702(7)	15.3066(9)
α/°	90	90
β/°	105.282(3)	105.612(5)
γ/°	90	90
V/Å^3	4526.4(16)	4209(2)
Z	4	4
D_c/g cm^{-3}	2.281	2.534
M	1605.75	1605.75
F(000)	2852	2736
T/ºC	25	25
$2\theta_{max}$/°	38.3	38.3
μ(Mo-*K*α)/mm^{-1}	4.034	4.341
No. refl. Measured	8322	7183
No. unique refl.	2957	2736
R_{int}	0.0291	0.0794
$R[F>2\sigma(F)]$[a]	0.0559	0.0901
$wR[F^2$, all refl.][b]	0.1796	0.2476
No. of refl. Used [$F>2\sigma$(F)]	2957	2736
No. of parameters	201	186
No. of restrains	169	168
S [c]	1.136	1.172
Max. residual electron density/e·Å^{-3}	0.69	1.85

	**0.9 GPa**	**1.0 GPa**
Chemical Formula	$C_{46}H_{16}PbAu_2F_{20}N_4$	$C_{46}H_{16}PbAu_2F_{20}N_4$
Crystal habit	Green prism	Green prism
Crystal size/mm	0.07x0.06x0.03	0.08x0.07x0.03
Crystal system	Monoclinic	Monoclinic
Space group	*Cc*	*Cc*
a/Å	11.3931(12)	10.9626(4)
b/Å	24.85(2)	25.416(7)
c/Å	15.2263(17)	15.3273(5)
α/°	90	90
β/°	105.695(11)	107.416(4)
γ/°	90	90
V/Å^3	4150(3)	4074.8(12)
Z	4	4
D_c/g cm^{-3}	2.570	2.617
M	1605.75	1605.75
F(000)	2960	2960
T/°C	25	25
$2\theta_{max}$/°	35.9	35.9
μ(Mo-$K\alpha$)/mm^{-1}	4.403	4.484
No. refl. Measured	6476	9492
No. unique refl.	2455	2577
R_{int}	0.0668	0.0568
$R[F>2\sigma(F)]$[a]	0.0776	0.0706
$wR[F^2$, all refl.][b]	0.2423	0.2328
No. of refl. Used [$F>2\sigma$(F)]	2455	2577
No. of parameters	201	206
No. of restrains	175	162
S [c]	1.053	1.189
Max. residual electron density/e·Å^{-3}	0.55	1.49

	**1.3 GPa**	**2.1 GPa**
Chemical Formula	$C_{46}H_{16}PbAu_2F_{20}N_4$	$C_{46}H_{16}PbAu_2F_{20}N_4$
Crystal habit	Green prism	Green prism
Crystal size/mm	0.07x0.06x0.03	0.08x0.07x0.03
Crystal system	Monoclinic	Monoclinic
Space group	*Cc*	*Cc*
a/Å	10.8530(3)	10.7511(5)
b/Å	25.557(8)	25.377(9)
c/Å	15.1439(6)	14.9000(6)
α/°	90	90
β/°	107.531(3)	107.645(5)
γ/°	90	90
V/Å^3	4005.4(12)	3873.9(13)
Z	4	4
D_c/g cm^{-3}	2.663	2.753
M	1605.75	1605.75
F(000)	2960	2960
T/ºC	25	25
$2\theta_{max}$/°	35.9	35.9
μ(Mo-*K*α)/mm^{-1}	4.562	4.717
No. refl. Measured	9074	10219
No. unique refl.	2489	2581
R_{int}	0.0408	0.0646
$R[F>2\sigma(F)]$[a]	0.0586	0.0507
$wR[F^2$, all refl.][b]	0.1641	0.1541
No. of refl. Used [$F>2\sigma(F)$]	2489	2581
No. of parameters	206	206
No. of restrains	162	162
S [c]	1.156	1.147
Max. residual electron density/e·Å^{-3}	1.08	1.13

Table S6. Crystallographic data for **1** between 100 and 298 K at ambient pressure.

	**100 K**	**200 K**
Chemical Formula	$C_{46}H_{16}PbAu_2F_{20}N_4$	$C_{46}H_{16}PbAu_2F_{20}N_4$
Crystal habit	Green prism	Green prism
Crystal size/mm	0.100 × 0.067 × 0.025	0.100 × 0.067 × 0.025
Crystal system	Monoclinic	Monoclinic
Space group	*Cc*	*Cc*
a/Å	11.6003(8)	11.6160(6)
b/Å	25.1273(17)	25.2465(14)
c/Å	15.6246(9)	15.7504(8)
α/°	90	90
β/°	105.933(2)	105.727(2)
γ/°	90	90
V/Å^3	4379.4(5)	4446.1(4)
Z	4	4
D_c/g cm^{-3}	2.435	2.399
M	1605.75	1605.75
F(000)	2960	2960
T/ºC	-173	-73
$2\theta_{max}$/°	55.8	55.8
μ(Mo-$K\alpha$)/mm^{-1}	10.645	10.485
No. refl. Measured	68095	47189
No. unique refl.	10159	10158
R_{int}	0.0298	0.0350
$R[F>2\sigma(F)]$[a]	0.0269	0.0665
$wR[F^2$, all refl.][b]	0.0660	0.0320
No. of refl. Used $[F>2\sigma(F)]$	10159	0.0779
No. of parameters	634	622
No. of restrains	227	269
S [c]	1.040	1.037
Max. residual electron density/e·Å^{-3}	1.59	1.28

	**285 K**
Chemical Formula	$C_{46}H_{16}PbAu_2F_{20}N_4$
Crystal habit	Green prism
Crystal size/mm	0.100 × 0.067 × 0.025
Crystal system	Monoclinic
Space group	*Cc*
a/Å	11.6283(7)
b/Å	25.3116(15)
c/Å	15.8823(8)
α/°	90
β/°	105.370(2)
γ/°	90
V/Å^3	4507.5(4)
Z	4
D_c/g cm^{-3}	2.366
M	1605.75
F(000)	2960
T/ºC	12
$2\theta_{max}$/°	56.0
μ(Mo-$K\alpha$)/mm^{-1}	10.343
No. refl. Measured	62611
No. unique refl.	10248
R_{int}	0.0391
$R[F>2\sigma(F)]$[a]	0.0369
$wR[F^2$, all refl.][b]	0.0867
No. of refl. Used [F>2σ(F)]	10248
No. of parameters	652
No. of restrains	233
S [c]	1.075
Max. residual electron density/e·Å^{-3}	1.33

II. Optical properties

1. UV-VIS ABSORPTION SPECTRA IN SOLUTION

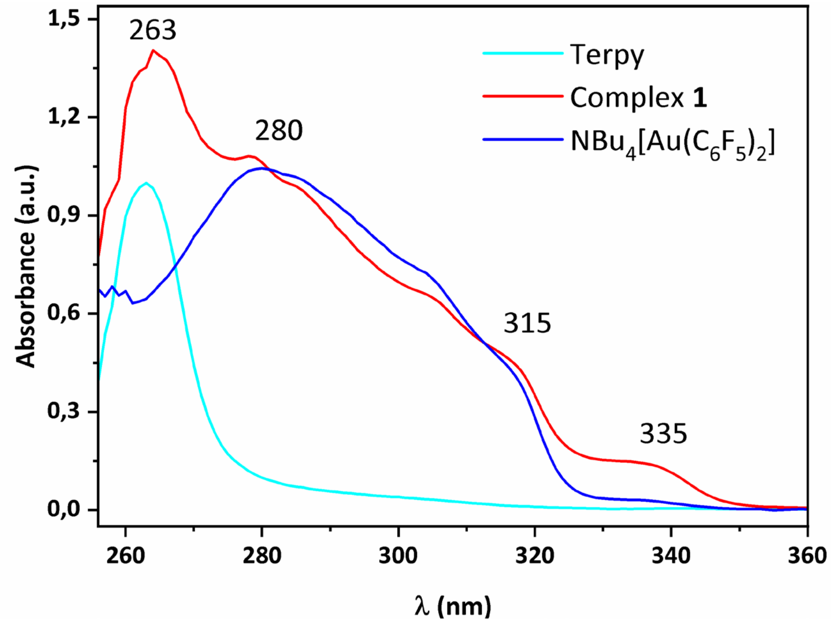

Figure S16. UV−vis absorption spectra in DMSO solution for complex **1** (red), gold(I) precursor $NBu_4[Au(C_6F_5)_2]$ (dark blue) and terpyridine ligand (light blue).

2. UV-VIS ABSORPTION SPECTRA IN SOLID STATE

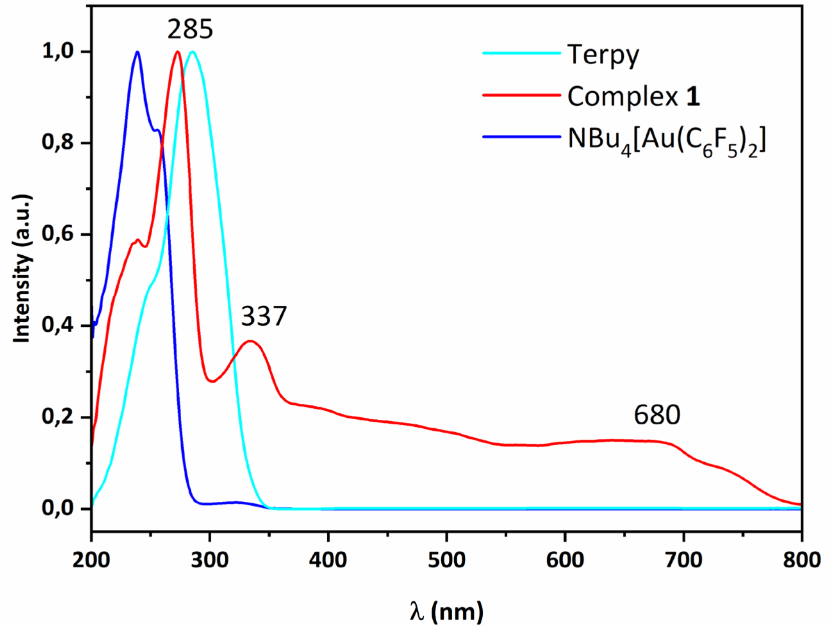

Figure S17. UV−vis absorption spectra in solid state for complex **1** (red), gold(I) precursor $NBu_4[Au(C_6F_5)_2]$ (dark blue) and terpyridine ligand (light blue).

3. PHOTOPHYSICAL PROPERTIES

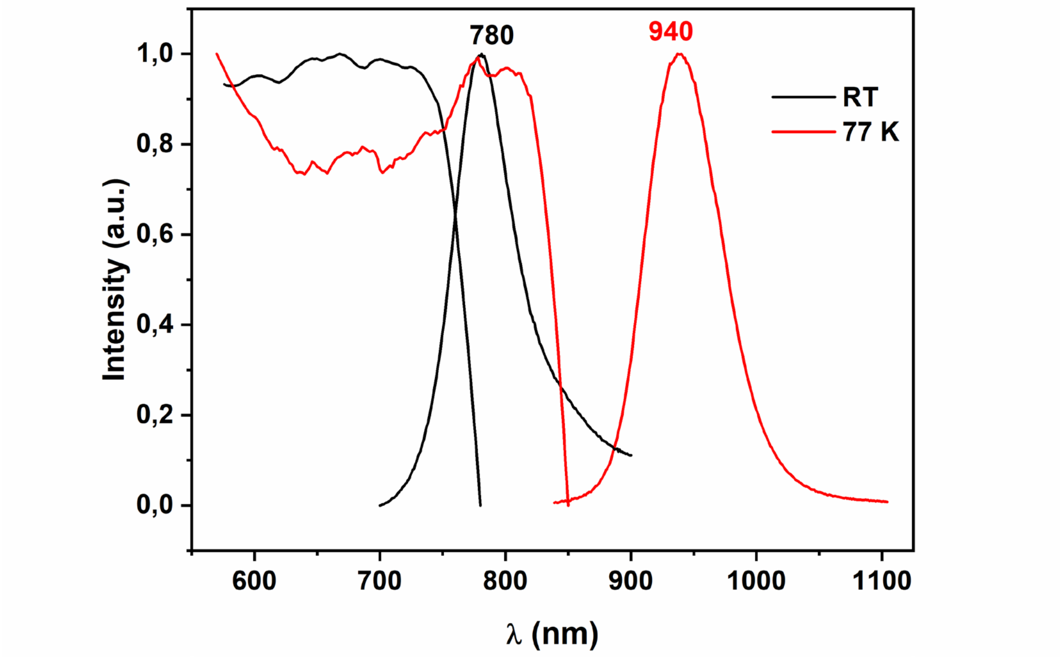

Figure S18. Excitation and emission spectra of complex **1** in solid state at room temperature (black) and 77 K (red).

III. Computational studies

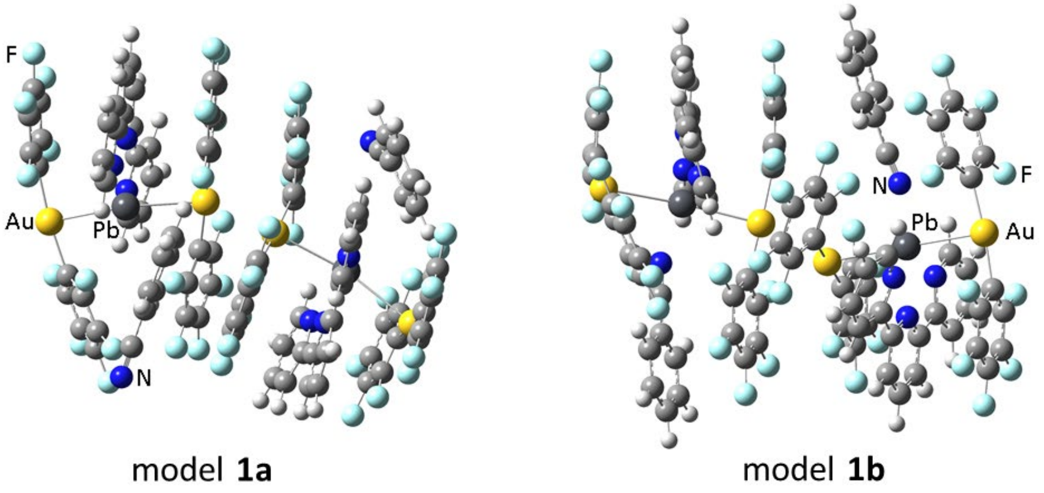

Figure S19. Model systems **1a** and **1b**.

1. ORBITALS INVOLVED IN MOST IMPORTANT TRANSITIONS FOR MODEL 1a

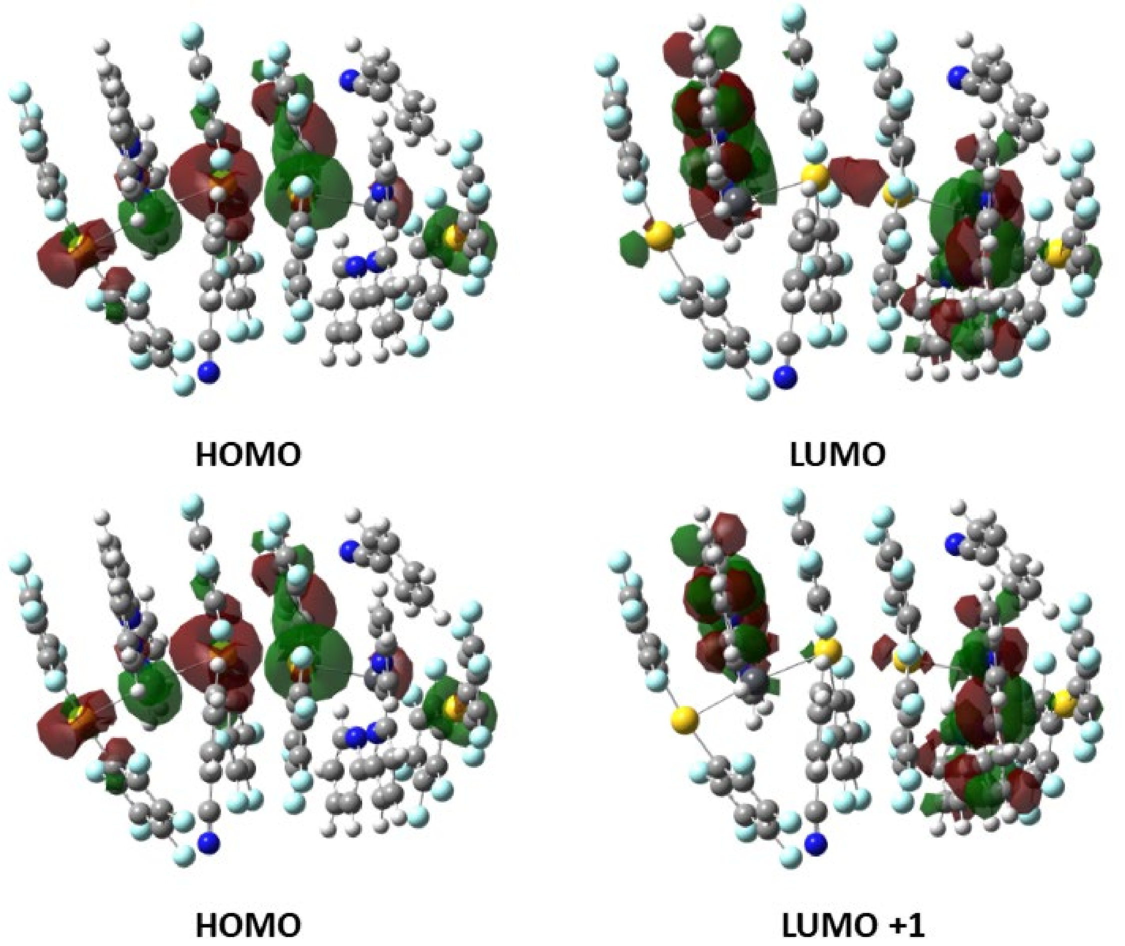

Figure S20. Molecular orbitals involved in the most important transitions calculated for the **1a** model.

2. ORBITALS INVOLVED IN MOST IMPORTANT TRANSITIONS FOR MODEL 1b

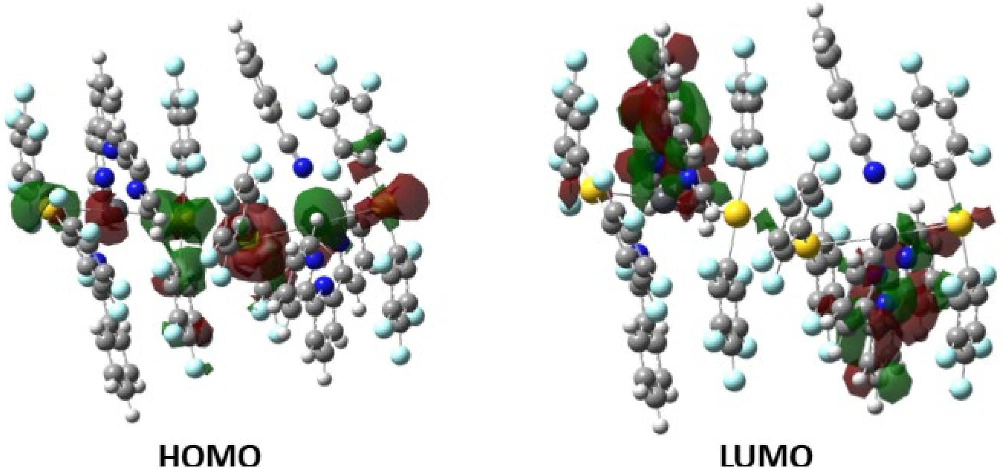

Figure S21. Molecular orbitals involved in the most important transitions calculated for the **1b** model.

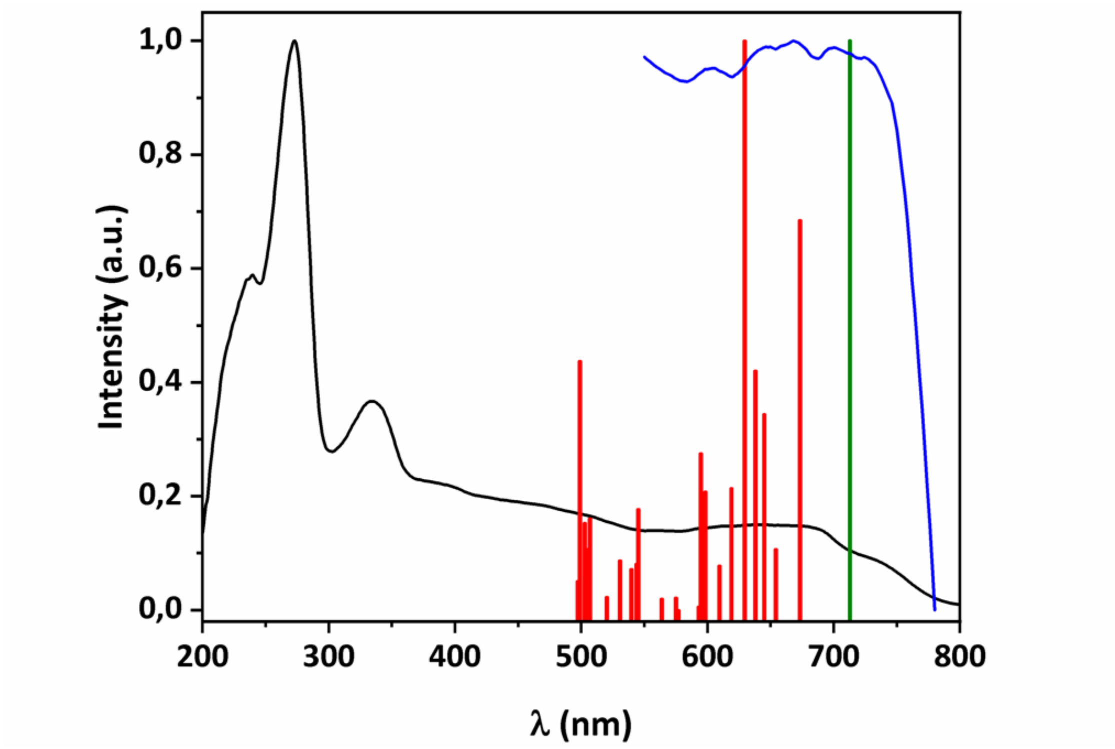

Figure S22. Comparison between solid-state UV-vis absorption spectra (black), experimental excitation spectra (blue), theoretical singlet-singlet excitations (red) calculated from model **1a** and theoretical singlet-triplet excitation (green) calculated from model **1b**.

Table S7. Population analysis (%) of the molecular orbitals involved in the most important transitions calculated for models **1a** and **1b**.

Model	Orbital	Au	C_6F_5	Pb	Terpy	Benzonitrile
**1a**	**LUMO+1**	5	5	6	83	1
	**LUMO**	10	8	14	69	0
	**HOMO**	60	24	10	3	1
**1b**	**LUMO**	6	7	8	79	0
	**HOMO**	58	27	10	4	1

3. 3-D ELECTRON LOCALIZATION FUNCTION (ELF) FOR MODELS 1a AND 1b

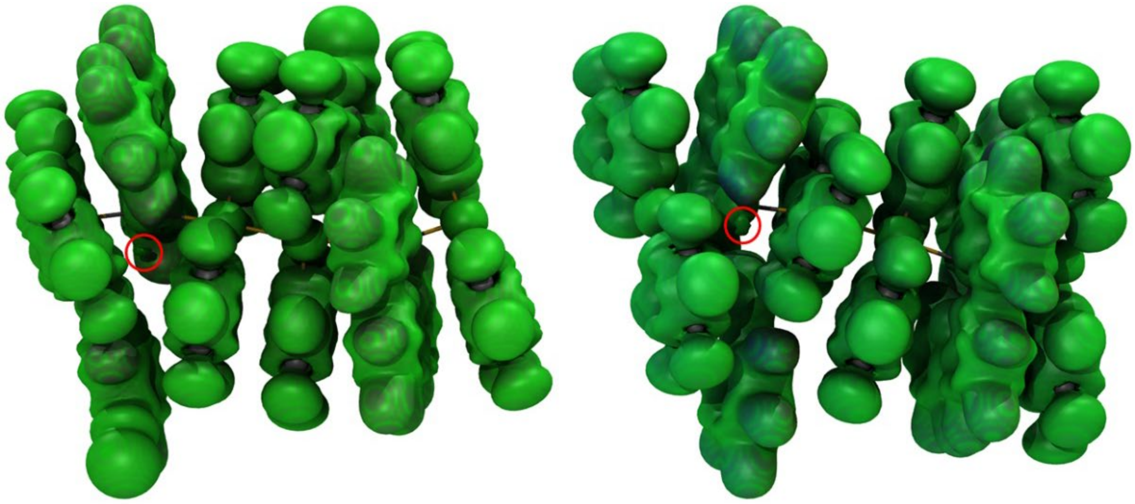

Figure S23. 3D-ELF plots of model systems **1a** ($\eta = 0.5$, left) and **1b** ($\eta = 0.4$, right). Lone electron pair probability for the Pb(II) centres can be localized (red circles).
